# Hybrid micelles containing methotrexate-conjugated polymer and co-loaded with microRNA-124 for rheumatoid arthritis therapy

**DOI:** 10.7150/thno.32268

**Published:** 2019-07-09

**Authors:** Fei Hao, Robert J Lee, Lihuang Zhong, Shiyan Dong, Chunmiao Yang, Lirong Teng, Qingfan Meng, Jiahui Lu, Jing Xie, Lesheng Teng

**Affiliations:** 1 School of Life Sciences, Jilin University, No.2699, Qianjin Street, Changchun130012, P.R. China; 2 College of Pharmacy, The Ohio State University, Columbus, 500 W 12th Ave, Columbus, OH 43210, USA

**Keywords:** polymeric hybrid micelles, folate receptor, methotrexate, microRNA-124, rheumatoid arthritis

## Abstract

**Purpose**: Methotrexate (MTX) is a first-line drug for rheumatoid arthritis (RA)therapy. However, MTX monotherapy often results in irreversible joint damage due to its slow onset of action and long duration. microRNA-124 (miR-124) has shown direct bone protection activity against RA. A co-delivery system for MTX and microRNA combination may provide therapeutic synergy.

**Methods**: Methotrexate-conjugated polymer hybrid micelles (M-PHMs) were prepared by self-assembly of two functional amphiphilic polymers (MTX-PEI-LA and mPEG-LA) at an optimized weight ratio. Incorporation of microRNA was achieved through electrostatic interactions between microRNA and cationic polymer MTX-PEI-LA. Cellular uptake, endosome escape, biodistribution, and therapeutic efficacy of M-PHMs/miR-124 complexes were investigated and evaluated in RAW264.7 cells and a rat adjuvant-induced arthritis (AIA) model.

**Results**: M-PHMs/miR-124 complexes exhibited folate receptor-mediated uptake in activated RAW264.7 cells. miR-124 was able to escape from the endosome and down-regulate nuclear factor of activated T cells cytoplasmic1 (NFATc1). M-PHMs/miR-124 complexes accumulated in inflamed joints of AIA rats and showed superior therapeutic efficacy through both anti-inflammatory effect and direct bone protective effect. Combination of miR-124 and MTX in these micelles induced disease remission.

**Conclusions**: M-PHMs/miR-124 was highly effective against RA through therapeutic synergy. Additional studies are warranted to further investigate its therapeutic potential and delineate its mechanisms of action.

## Introduction

Rheumatoid arthritis (RA) is a complex and incurable chronic autoimmune disease with multiple mechanisms of pathogeneses 1, 2. Disease-modifying antirheumatic drugs (DMARDs) are used for the treatment of RA. However, a monotherapy usually cannot achieve sustained and complete disease remission 3, 4. Accordingly, new treatments are urgently needed 5. Combination therapy of DMARDs and biomacromolecules such as siRNA 6, 7, microRNA 8, 9, and monoclonal antibody 10, 11 is a promising approach 4, 12. Methotrexate (MTX) is a frequently used DMARD and as the anchor drug for RA treatment 13, 14. However, MTX monotherapy often results in drug resistance and irreversible joint damage due to its slow onset of action and long duration 15-18. MTX combination with biomacromolecules therapy is under intense investigation 19-22. MicroRNAs are highly conserved sequences and post-transcriptional regulators of gene expression and are emerging as a novel therapeutic modality 8, 9, 23. Each microRNA usually consists of 19-24 nucleotides and can simultaneously regulate multiple targets associated with disease 9, 24. MicroRNA-124 (miR-124) recently has been identified as an RA-associated microRNA and a potential therapeutic target for RA treatment 25, 26. pre-miR-124 injected into the joints of adjuvant-induced arthritis (AIA) rat model has been shown to effectively reduce bone destruction and osteoclast genesis 27. MTX and miR-124 combination therapy maybe more effective against RA 28.

Nanoparticle-based therapy of RA has received much attention in recent years 29-31. To increase the stability, reduce the off-target effects, and efficiently deliver both miR-124 and MTX to the specific targeting tissue, it is desirable to develop a targeted nanoparticle delivery system 31. Activated macrophages have recently been reported as a promising target for RA nanotherapeutics. Receptors including folate receptor beta (FRβ), scavenger receptor (SR), and hyaluronic acid receptor (CD44) are elevated in these cells 32-38. Since FRβ is not expressed in most normal tissues, drug carriers targeting FRβ can be used to deliver drugs into these cells 32, 33, 39-47. However, conventional FR-targeted delivery systems are often composed of distinct functional components for targeting and therapeutic activity 46. Being able to combine both functions serves to simplify the composition of the delivery system, therefore, is desirable 31.

Polymer-drug conjugates have been used to increase drug solubility, alter drug pharmacokinetics, enhance drug therapeutic efficacy, and reduce systemic side-effects 48-50. Furthermore, the drugs conjugated to polymers had great potential to achieve both therapeutic and targeted functions. Polymer-drug conjugates can form part of a multifunctional carrier for combination therapy 50, 51. As a folic acid (FA) analog, MTX conjugated to polymers have recently been shown to target folate receptors 52-55. MTX attached to generation 5 dendrimer (G5-MTX) via amide bond not only induced the cytotoxicity but greatly enhanced the affinity for folate receptor alpha (FRα) 53, 54. Multivalent MTX was shown to bind FRα 56. Recent studies showed that the amino groups of MTX play a key role in targeting FRα 57. Most recently, studies have found that G5-PAMAM-FA-MTX has a stronger FRβ targeting effect compared to G5-PAMAM-FA 42. Nakashima-Matsushita et al. reported that MTX could be transported to the synovial macrophages through FRβ 58. Qi et al. further demonstrated that G5-MTX was taken up by activated macrophages overexpressing FRβ and had a similar anti-inflammatory effect as MTX at an equivalent dose 59. Inspired by these findings, we hypothesized that MTX can be used to achieve efficient macrophage targeting and to exert therapeutic activity for RA.

Polyethylenimine(PEI) is a well-known non-viral cationic carrier for the delivery of nucleic acids, including siRNA and microRNA 60-62. In addition, PEI can be used to construct a polymer-drug conjugate due to its abundance of amine functional group. PEI-drug conjugates can be further used for the delivery of oligonucleotides. Dong et al. prepared pre-functionalized polymer-drug conjugates for combination therapy of siRNA and chemotherapy drugs by coupling doxorubicin (DOX) and PEI 63. Xu et al. also synthesized PEI-DOX for co-delivery of Bcl2 siRNA and DOX 64. Both co-delivery systems exhibited a synergistic and enhanced antitumor efficacy.

**Scheme 1:**
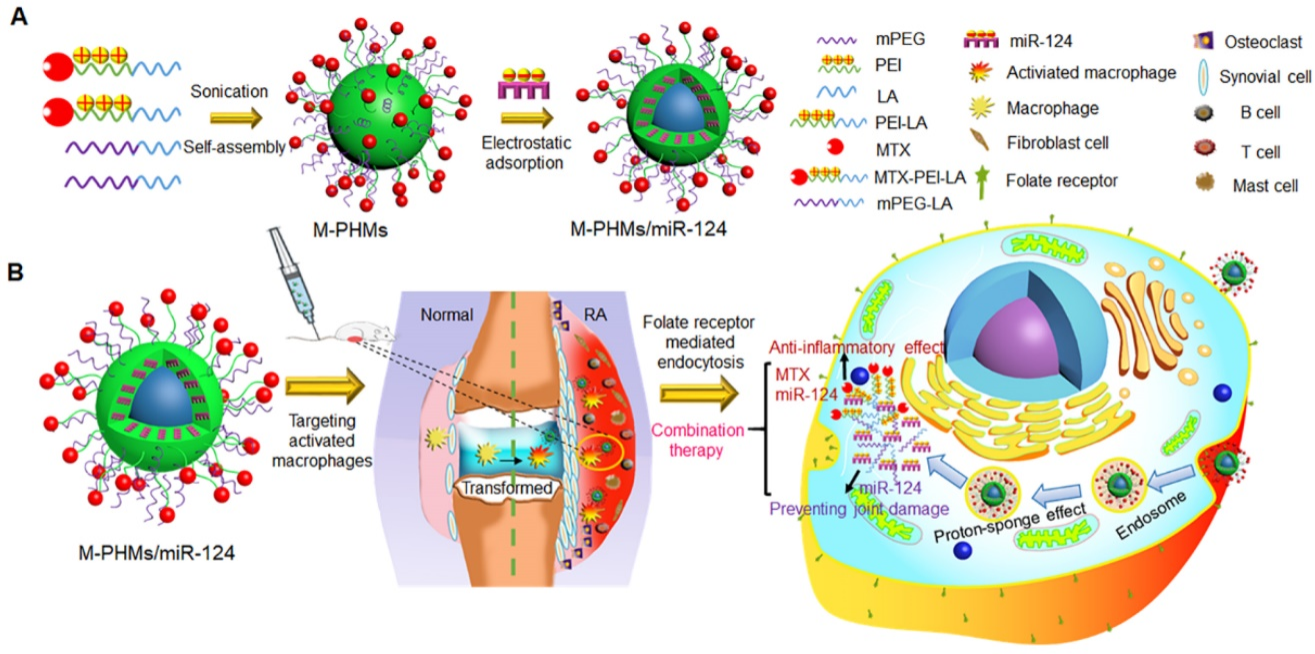
Schematic representation of MTX-conjugated polymer hybrid micelles co-loaded with miR-124 (M-PHMs/miR-124) preparation and combination therapy of M-PHMs/miR-124 in RA. (A) The M-PHMs were formed by the self-assembly of MTX-PEI-LA and mPEG-LA. miR-124 was then loaded through electrostatic interactions with the micelles. (B) M-PHMs/miR-124 targeted to the folate receptor (FR) on activated macrophages in RA joints and released MTX conjugates and miR-124 into the cytoplasm by the proton-sponge effect. The combination therapy had both anti-inflammatory effect and joint protective effect.

In our previous study, it was shown that fatty acid, including oleic acid and linoleic acid, modified PEI exhibited a higher transfection efficiency for oligonucleotides and reduced cytotoxicity for normal cells 65, 66. To achieve efficient co-delivery and combination therapy of microRNA and MTX with low carrier toxicity, MTX was first attached to linoleic acid modified branched PEI (MTX-PEI-LA). MTX-conjugated polymer hybrid micelles (M-PHMs) were self-assembled from a combination of MTX-PEI-LA and linoleic acid-modified methoxy-polyethylene glycol (mPEG-LA) and then complexed to miR-124, as illustrated in **Scheme 1A**. We proposed that M-PHMs/miR-124 could specifically target the activated macrophages in RA joints, successfully release the payloads into the cytoplasm, and achieve the combination therapy as shown in **Scheme 1B.**

## Results and Discussion

### Synthesis and characterization of MTX-PEI-LA and mPEG-LA

**Figure 1:**
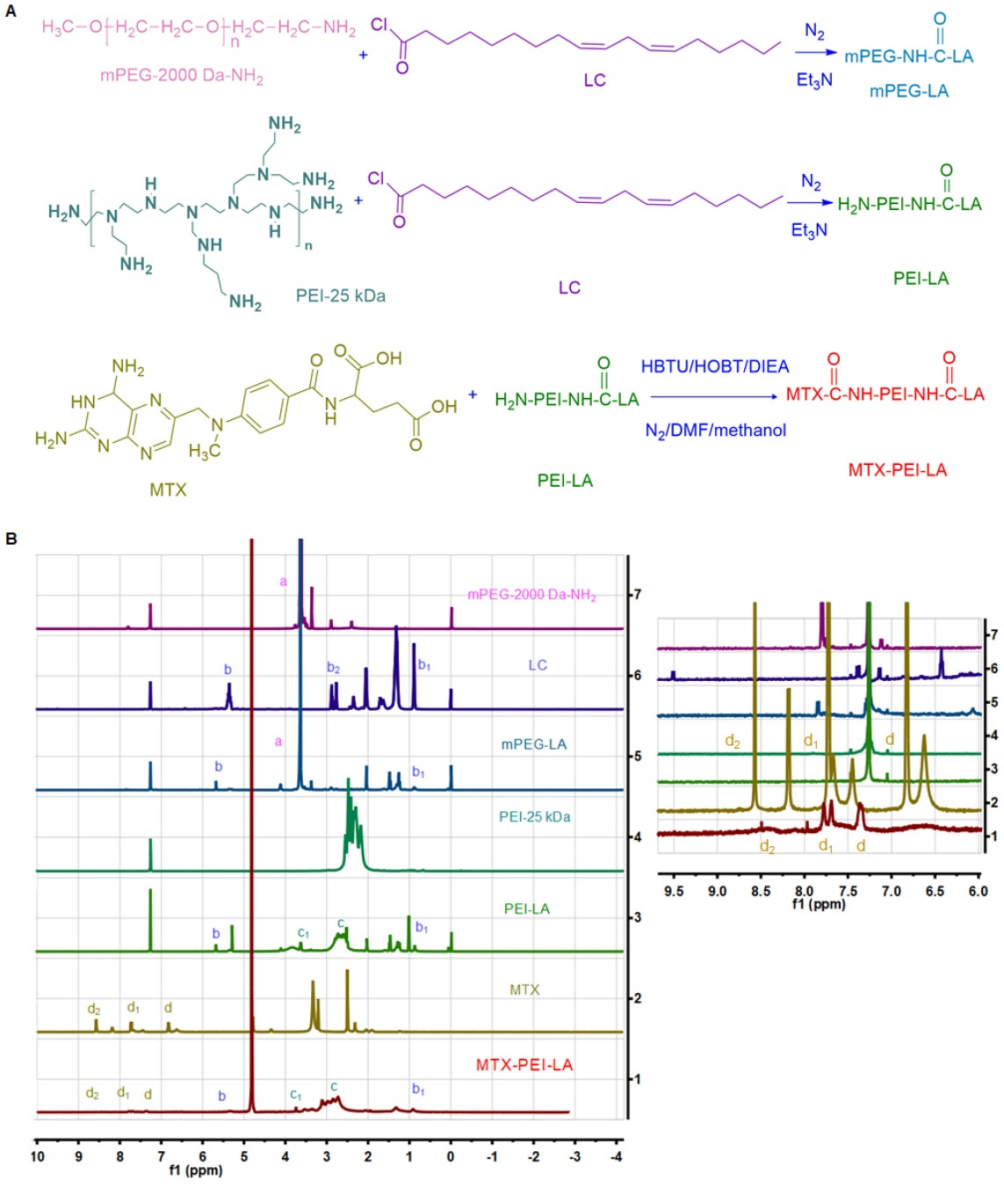
The chemical synthesis procedure and ^1^H NMR spectrum of polymer conjugates. (A) Linoleyl chloride (LC) was first conjugated to the mPEG-NH_2_ and PEI through the reaction of an acid chloride with an amine. MTX was then conjugated to the H_2_N-PEI-LA through the acid and amine condensation reaction. (B) ^1^H NMR spectrum (500 MHz) of mPEG-2000 Da-NH_2_, LC, mPEG-LA, PEI-25 kDa, PEI-LA, MTX, MTX-PEI-LA. mPEG-2000 Da-NH_2_, LC, PEI-25 kDa, mPEG-LA, and PEI-LA were dissolved in deuterated chloroform (CDCl_3_). MTX was dissolved in deuterated dimethyl sulfoxide (DMSO-d_6_). MTX-PEI-LA was dissolved in deuterated water (D_2_O).

The synthesis procedure was shown in **Figure 1A** and chemical structures of the two amphiphilic polymers (MTX-PEI-LA and mPEG-LA) were confirmed by ^1^H NMR and Fourier-transform infrared (FT-IR) spectra (**Figure 1B and Figure S1**). mPEG-2000 Da-NH_2_ had a prominent peak at 3.62 ppm for methylene (a, 1 alpha-O-C and 1beta-O-C). Linoleyl chloride (LC) had prominent peaks at 5.35 ppm for 1-ethylene (b, 1-(C=C) or 1-C), 0.89 ppm for methyl (b1) and characteristic peaks at 2.88 ppm for methylene (b2, 1alpha-C(C=0) Cl) in deuterated chloroform (CDCl_3_). The characteristic peaks of PEI-25 kDa were also observed at 1.8-2.8 ppm (c) in CDCl_3_. In the ^1^H NMR spectra of mPEG-LA and PEI-LA (soluble in CDCl_3_), the prominent peaks of mPEG-2000 Da-NH_2_ (a), LC (b, b_1_) and PEI-25 kDa (c) can be also found. However, the characteristic peak (b_2_) of LC disappeared in the spectra of mPEG-LA and PEI-LA. This indicated that a chemical reaction had occurred and LC was successfully conjugated to mPEG-2000 Da-NH_2_ and PEI-25 kDa. The characteristic peaks of MTX in ^1^H NMR spectra were observed at 6.76 ppm (d) and 7.64 ppm (d_1_) for 1-benzene, and 8.57 ppm (d_2_) for 2-pyrazine in deuterated dimethyl sulfoxide (DMSO-d_6_). In the ^1^H NMR spectra of MTX-PEI-LA conjugates (dissolved in deuterated water(D2O)), it not only contained the characteristic peaks of PEI-25 kDa (c), LC (b, b_1_,) but also the characteristic signals of MTX (d, d_1_, d_2_). MTX was successfully conjugated to PEI-LA. The characteristic peaks of aromatic protons in MTX conjugated with PEI-LA (d, δ=7.34; d_1_, δ=7.71; d_2_, δ=8.47) was a little offset. The peak shift may be induced by the reaction of MTX with PEI-LA. In FT-IR spectra, new peaks could be found at 3300-3500 cm^-1^ in mPEG-LA (**Figure S1A**) and 3270 cm^-1^ (**Figure S1B**) in PEI-LA due to the amide bond generation. As shown in **Figure S1B**, the characteristic peaks of the carboxyl group in MTX at 1602 cm^-1^ and 1646 cm^-1^ disappeared in MTX-PEI-LA 35. All of this further confirmed that the MTX-PEI-LA and mPEG-LA were successfully synthesized. MTX was conjugated to PEI-LA by an amide for several reasons. First of all, serum-stable amide provides greater stability in the blood 53. Secondly, the MTX-conjugated polymers could be applied as a potential long-circulating formulation according to its stability. Finally, MTX-conjugated polymers may alter the pharmacokinetics and reduce the systemic toxicity of MTX. The absorption peak of MTX in MTX-PEI-LA was confirmed at 303 nm (**Figure S2**) and the drug loading efficiency were calculated to be 15.6%.

### Formulation optimization for M-PHMs and M-PHMs/miR-124

M-PHMs were prepared by the self-assembly of MTX-PEI-LA and mPEG-LA. The properties of M-PHMs especially the cationic charge can be easily adjusted by changing the weight percentage of MTX-PEI-LA. As shown in **Table S1**, when the percentage of MTX-PEI-LA increased, the zeta potential of M-PHMs changed from negative to positive charge and the average size of M-PHMs also gradually increased. The particle sizes of M-PHMs with 5%, 10% and 20% of MTX-PEI-LA were all under 200 nm and had a narrow particle size distribution and low polydispersity index (PDI) (PDI < 0.25). Bovine serum albumin (BSA) precipitation assay was further conducted to assess the colloidal stability of M-PHMs with different percentages of MTX-PEI-LA in serum. As indicated in **Figure S3**, M-PHMs with 2% and 5% MTX-PEI-LA had a lower precipitation rate compared with M-PHMs with higher weight percentages of MTX-PEI-LA. The lower precipitation rate of M-PHMs demonstrated better stability in blood. In comparison with the M-PHMs (2% MTX-PEI-LA) with a negative zeta potential (-2.67 mV), M-PHMs (5% MTX-PEI-LA) with an appropriate positive zeta potential (7.57 mV) had a potential ability to form complexes with micro-RNA through electrostatic interactions. In summary, we chose to prepare M-PHMs composed of 5% MTX-PEI-LA and 95% mPEG-LA. In order to better characterize the M-PHMs properties, the polymer hybrid micelles (PHMs) without MTX conjugation were also prepared. The PHMs were comprised of PEI-LA and mPEG-LA and were set as a negative control for M-PHMs with potential targeted and therapeutic dual functions. The weight percentage of PEI-LA in PHMs was also set as 5%. M-PHMs or PHMs referred in all of the following studies both represented a composition of 5% MTX-PEI-LA or 5% PEI-LA.

**Figure 2:**
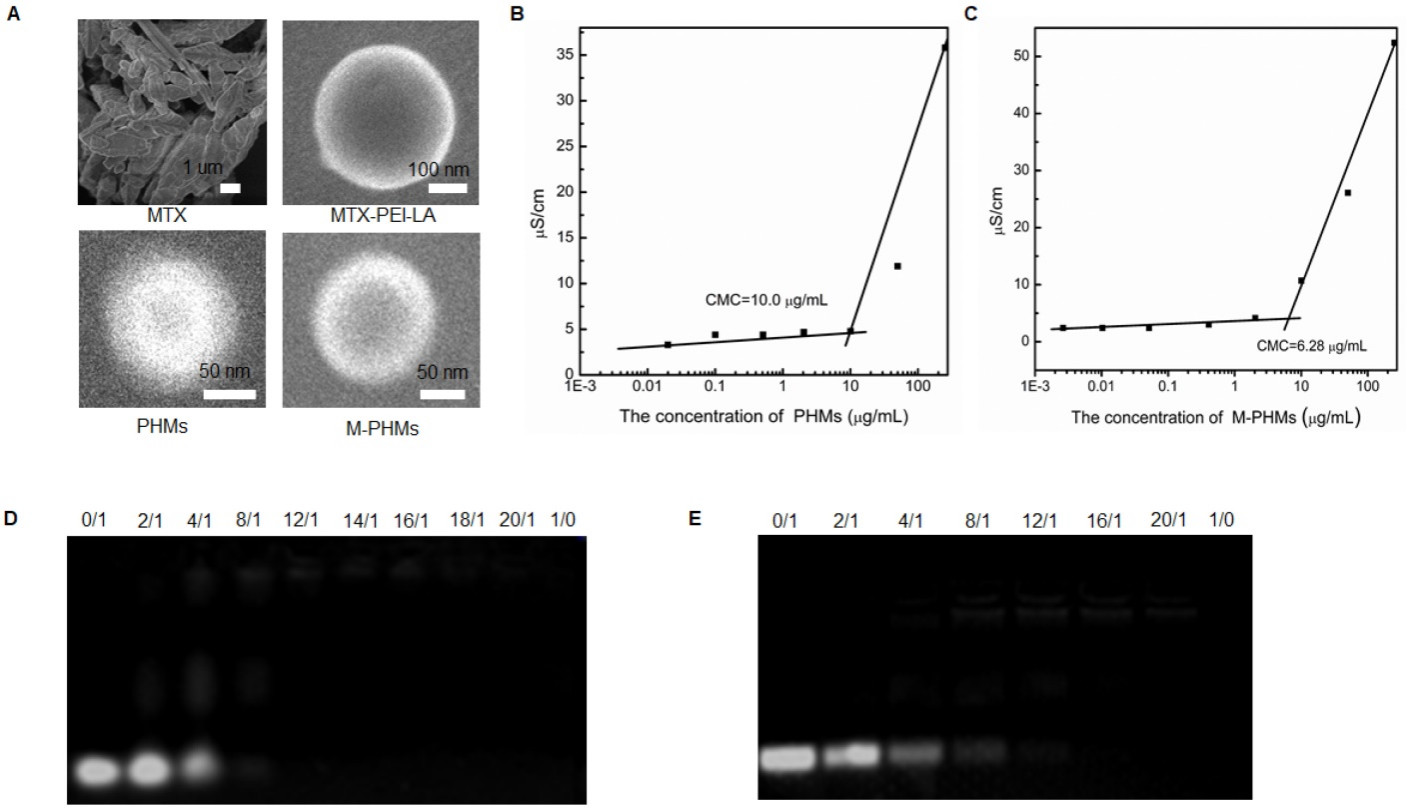
Characterization of PHMs and M-PHMs and their ability to complex miR-124. (A) The morphology of MTX, MTX-PEI-LA, PHMs, M-PHMs was obtained by field emission scanning electron microscopy (FESEM). The critical micelle concentration (CMC) of PHMs (B) and M-PHMs (C) was then obtained by measuring conductivity. The ability of PHMs (D), M-PHMs (E) to complex miR-124 was investigated by agarose gel electrophoresis. Nitrogen to phosphate ratios (N/P) of PHMs or M-PHMs to miR-124 varied from 0/1 to 20/1. N/P of 0/1 referred to the unencapsulated free miR-124. N/P of 1/0 referred to the vectors without miR-124.

The morphology of MTX, MTX-PEI-LA, PHMs, and M-PHMs was further visualized by field emission scanning electron microscopy (FESEM) (**Figure 2A**). Compared with the crystal packing of free MTX, both MTX-PEI-LA, PHMs, and M-PHM formed uniform spherical particles in water. MTX-PEI-LA had a larger particle size than the M-PHMs and PHMs. Unlike the smooth surface of MTX-PEI-LA micelles, PHM and M-PHMs had a rough surface which may be caused by the polyethylene glycol (PEG) layer on the surface of nanoparticles due to its hydrophilicity. The critical micelle concentration (CMC) of PHMs (**Figure 2B**) and M-PHMs (**Figure 2C**) were 10.0 µg/mL and 6.28 µg/mL, respectively. M-PHMs and PHMs both had a lower CMC and may have good stability in blood circulation. Then the PHMs/miR-124 or M-PHMs/miR-124 complexes formed at differing nitrogen to phosphate ratios (N/P) were analyzed by 2% agarose gel electrophoresis. The N/P ratios of PHMs or M-PHMs to miR-124 were calculated based on the molar ratio of the N atoms in PEI-LA or MTX-PEI-LA composed of micelles (PHMs or M-PHM) to the negatively charged P groups of the miR-124 molecule. In PEI, each unit has a molecular weight of 43 and contains one N. In miR-124, each unit weight of 330 contains one P. In this study, PEI had been modified by LA and MTX. The degree of functionalization with PEI with LA and MTX was calculated to be 12 and 15, respectively, based on the ^1^HNMR of PEI-LA and measured MTX concentration in MTX-PEI-LA. Thus, a molecular weight of 48.8 in PEI-LA and a molecular weight of 60.5 in MTX-PEI-LA contains one N, respectively. Based on the defined ratio of N/P, the weight of PEI-LA and MTX-PEI-LA required to complex a certain amount of miR-124 could be calculated. According to the weight percentages of PEI-LA and MTX-PEI-LA in the compositions of PHMs and M-PHMs, the corresponding weights of M-PHMs and PHMs required for a certain N/P ratio were calculated. As shown in **Figure 2D**, PHMs could successfully condense miR-124 with an N/P ratio of 12. When the N/P ratio was 16 or greater, M-PHMs could also completely condense miR-124 (**Figure 2E**). The MTX-PEI-LA or PEI-LA with a positive charge density in M-PHMs or PHMs is capable of having strong electrostatic interaction with the negatively charged miR-124. The size, zeta potential and PDI of the PHMs/miR-124 complexes (N/P=12) or M-PHMs/miR-124 complexes (N/P=16) were listed in **Table S2**. The size, zeta potential and PDI of PHMs/miR-124 were 112.7±3.9 nm, 4.77±1.85 mV, and 0.202±0.001 (n=3). The size, zeta potential and PDI of M-PHMs/miR-124 were 116.6±1.1 nm, -4.66±0.20 mV, and 0.217±0.008 (n=3), respectively. M-PHMs/miR-124 complexes had a smaller particle size than M-PHMs. This may be due to a more compact and stable structure of M-PHMs/miR-124 complexes after loaded with miR-124 by electrostatic interaction.

### *In vitro* FAM-miR-124 release profile of M-PHMs/FAM-miR-124

In the FAM-miR-124 release profile (**Figure S4**), we found that M-PHMs had a slow and controlled release of FAM-miR-124 in phosphate buffer saline (PBS, pH=7.4). The cumulative release of FAM-miR-124 loaded in M-PHMs/FAM-miR-124 complexes was less than 25% within 48 h. In the first 2 h, the FAM-miR-124 in M-PHMs/FAM-miR-124 complexes had a rapid release and the cumulative release was up to 10%. This might be caused by the FAM-miR-124 adsorbed on the outer surface of the M-PHMs. At 10 h, the release of FAM-miR-124 reached a maximum. A strong interaction of FAM-miR-124 with the cationic carrier in M-PHMs may play an important role in the slow release of FAM-miR-124. The results also indicated high stability of the M-PHMs/FAM-miR-124 complexes in PBS. We also studied the release of MTX in M-PHMs. Consistent with a previous study, no free MTX was detected within 72 h 55, 67. Since MTX was conjugated to the PEI-LA with a stable amide bond, MTX was not released in the PBS release medium at pH=7.4.

### FR-mediated cell uptake of M-PHMs/Cy3-miR-124 in lipopolysaccharide (LPS)-induced RAW 264.7 cells

**Figure 3:**
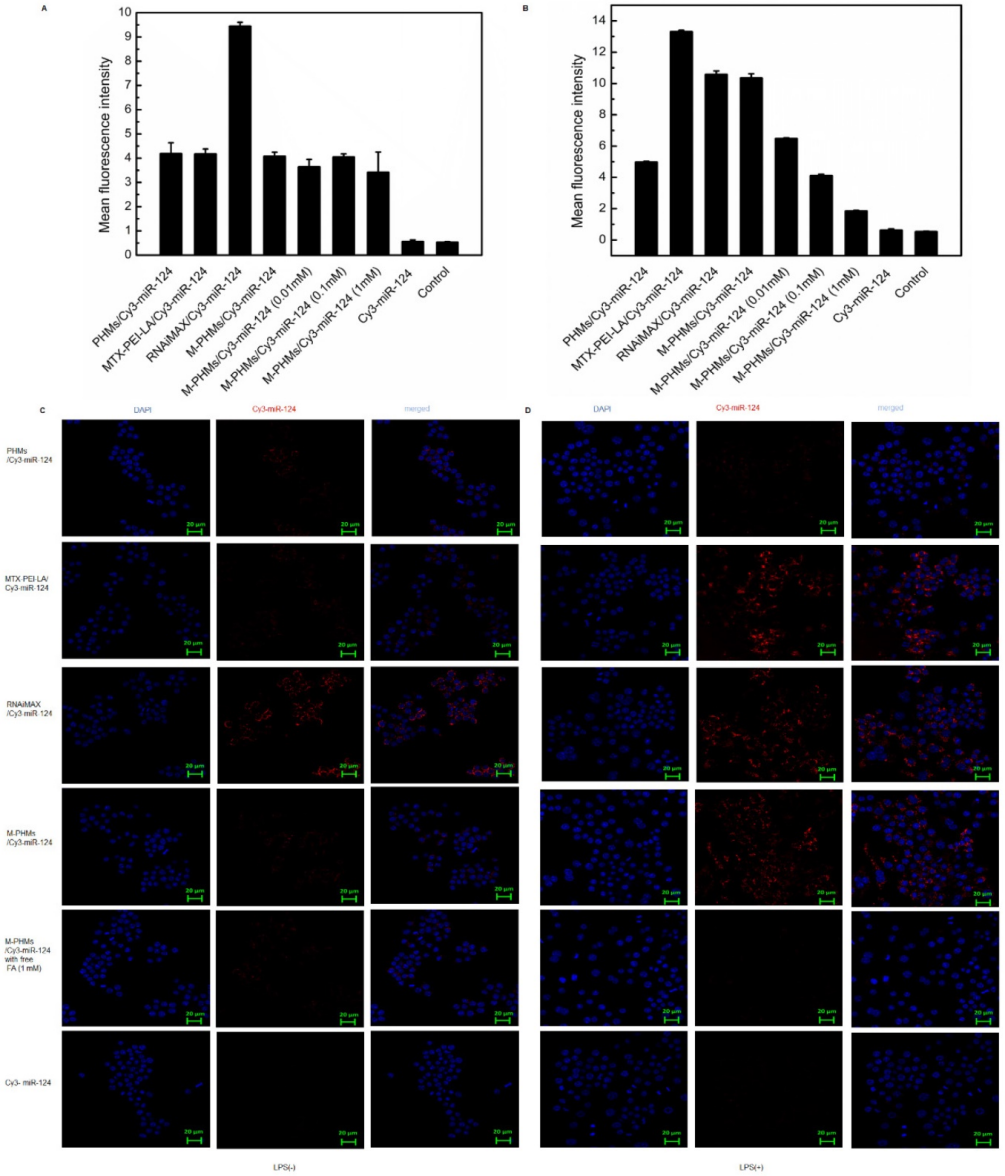
Cellular uptake of M-PHMs/Cy3-miR-124* in vitro*. The RAW 264.7 cells were either activated by lipopolysaccharide (LPS) (+) or not (-). Flow cytometry was first used for quantitative analysis cell uptake of M-PHMs/Cy3-miR-124. RAW 264.7 cells activated by LPS (-) (A) or LPS (+) (B) were incubated with PHMs/Cy3-miR-124, MTX-PEI-LA/Cy3-miR-124, RNAiMAX/Cy3-miR-124, M-PHMs/Cy3-miR-124, or Cy3-miR-124 for 4 h at 37 °C. Free folic acid (FA) at increasing concentrations (0.01 mM, 0.1 mM, 1 mM) was added 1 h before M-PHMs/Cy3-miR-124 (100 nM) was added to the cells. RNAiMAX was commercially available and set as a positive control for miR-124 delivery. The values were mean ± SEM (n = 3). Confocal micrographs of RAW 264.7cells with LPS (-) (C) and LPS (+) (D) were then obtained after being incubated with MTX-PEI-LA/Cy3-miR-124, RNAiMAX/Cy3-miR-124, M-PHMs/Cy3-miR-124, and PHMs/Cy3-miR124 for 4 h at 37 °C. Free FA was also added 1 h before the addition of M-PHMs/Cy3-miR-124 (100 nM) to the cells. Green Bar in the images was 20 μm.

The murine macrophage cell line RAW 264.7 was induced by LPS (1 µg/mL) to obtain activated macrophage-like cells overexpressing FRβ *in vitro* 45. Cellular uptake mediated by the FR was first investigated and quantified by flow cytometry and further visualized by confocal microscopy (**Figure 3**). MTX-PEI-LA, RNAiMAX, M-PHMs, and PHMs were loaded with Cy3-miR-124 with red fluorescence. RNAiMAX was commercially available and set as a positive control for miR-124 delivery *in vitro*. According to the quantitative flow cytometric analysis, the cellular uptake intensity of M-PHMs/Cy3-miR-124 and MTX-PEI-LA/Cy3-miR-124 was depending on the activation status of macrophages. A much higher mean fluorescence intensity was found in M-PHMs/Cy3-miR-124 and MTX-PEI-LA/Cy3-miR-124 treated group compared with PHMs/Cy3-miR-124 without MTX conjugated in LPS-induced RAW 264.7 cells. The mean fluorescence intensity of LPS-induced RAW 264.7 cells treated with M-PHMs/Cy3-miR-124 was as strong as those treated with RNAiMAX/Cy3-miR-124. Simultaneously, the mean fluorescence intensity could be gradually decreased in activated macrophages pre-incubated with increasing FA concentrations (0.01 mM, 0.1 mM, 1 mM). MTX-PEI-LA/Cy3-miR-124 had a higher cellular uptake in LPS-induced RAW 264.7 cells than M-PHMs/Cy3-miR-124. This may be related to its higher positive charge. However, there was almost no significant difference in cellular uptake of M-PHMs/Cy3-miR-124 and MTX-PEI-LA/Cy3-miR-124 compared with PHMs/Cy3-miR-124 in non-activated macrophages (**Figure 3A**). Meanwhile, the mean fluorescence intensity of the cells treated with MTX-PEI-LA/Cy3-miR-124 and M-PHMs/Cy3-miR-124 was both low in RAW 264.7 cells without LPS induction. The pre-incubation of FA had no effect on the cellular uptake of M-PHMs/Cy3-miR-124 in RAW 264.7 cells without LPS induction. The mean fluorescence intensity of RAW 264.7 cells treated with RNAiMAX/Cy3-miR-124 or PHMs/Cy3-miR-124, with or without LPS induction, was almost the same. The same results were observed in the confocal results. As was shown in** Figure 3C-D**, PHMs/Cy3-miR-124, MTX-PEI-LA/Cy3-miR-124, and M-PHMs/Cy3-miR-124 had lower uptake and lower red fluorescence intensity in non-activated macrophages. However, the red fluorescence intensity in the cytoplasm of activated macrophages treated with MTX-PEI-LA/Cy3-miR-124 or M-PHMs/Cy3-miR-124 was markedly stronger than those of cells treated with naked Cy3-miR-124 or PHMs/Cy3-miR-124. Furthermore, the fluorescence intensity of cells treated with M-PHMs/Cy3-miR-124 was significantly reduced and almost undetectable in cells pre-incubated with free FA (1 mM). Meanwhile, the red fluorescence intensity in non-activated macrophages treated with M-PHMs/Cy3-miR-124 was almost undetectable and had no significant change with preincubation with free FA (1 mM). These results demonstrated that the cellular uptake of M-PHMs/Cy3-miR-124 could be greatly increased in the activated macrophages and be inhibited by adding free FA. M-PHMs/Cy3-miR-124 entered FR-highly expressed macrophages mainly by FR-mediated endocytosis and had a promising FR targeting ability.

### Endosome escape of M-PHMs/FAM-miR-124 in LPS-induced RAW 264.7 cells

**Figure 4:**
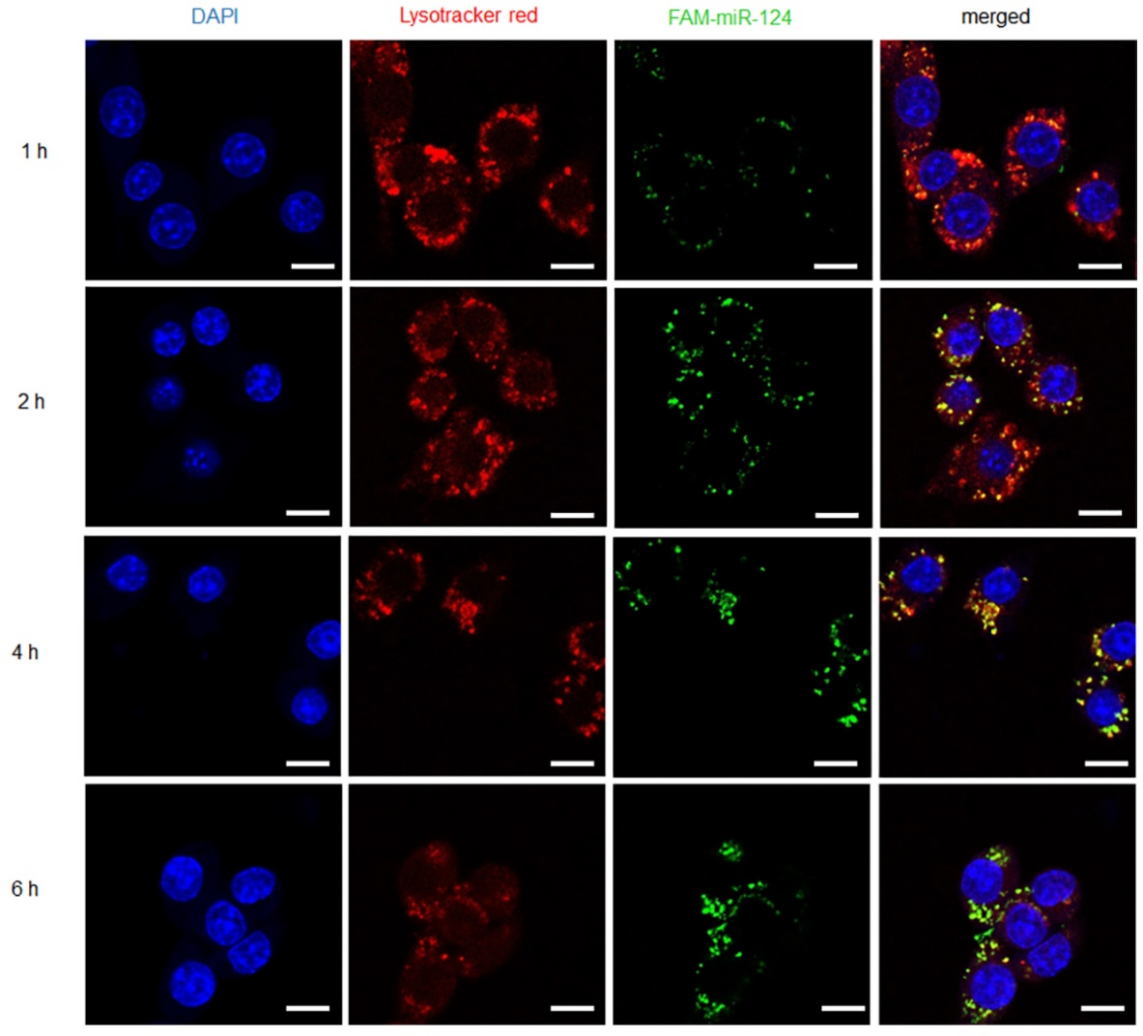
Internalization and endosome escape of M-PHMs/FAM-miR-124 complexes in activated RAW 264.7 cells. Internalization process and escape of M-PHMs loaded with FAM-miR-124 (green) from endosomes (red) of activated macrophages was visualized by confocal laser scanning microscopy (CLSM) at 1, 2, 4, and 6 h. The scale bar in the images was 10 μm.

The fluorescence-labeled complexes were efficiently taken up by RAW 264.7 macrophages with LPS activation. However, the drug release of M-PHMs/FAM-miR-124 at the subcellular level still remained unclear. Thus, another fluorescent reagent -Lyso Tracker™ Red DND-99 was used to label endosomes and investigate whether FAM-miR-124 could escape from the endosome and be released into the cytoplasm. As shown in** Figure 4**, the cellular uptake of M-PHMs/FAM-miR-124 was increased as the incubation time increased and the green fluorescence intensity reached the maximum value at 4 h. The green and red signals started to overlap at 1 h, indicating that M-PHMs/FAM-miR-124 began to be internalized into endosomes. At 2 h, the overlap of green and red signals started to increase. At 4 h, the overlap of green and red signals (orange color) reached a maximum, implying that FAM-miR-124 was all located in the endosome. At 6 h, the green signal was clearly isolated from the red signal, indicating that FAM-miR-124 had escaped from the endosome and was successfully released into the cytosol. The endosome rupture may be due to the proton sponge effect of PEI in M-PHMs 60, 61. All in all, miR-124 in M-PHMs/miR-124 could be efficiently internalized into the cells with high FR expression and successfully released into the cytoplasm.

### Inhibition of nuclear factor of activated T cells cytoplasmic 1 (NFATc1) expression and cell cytotoxicity of M-PHMs/miR-124

NFATc1 is the key factor for osteoclast differentiation and is induced by receptor activator of nuclear factor-κB (NF-κB) ligand (RANKL) 69, 70. miR-124 has been reported to directly target NFATc1 mRNAs, reduce NFATc1 expression levels, suppress the differentiation of osteoclast differentiation, and finally inhibit the progression of RA 27. Therefore, the gene silencing efficiency of miR-124 can be assessed by measuring the expression level of NFATc1. As shown in **Figure S5**, RAW 264.7 cells stimulated with RANKL and treated with M-PHMs/miR-124 and MTX-PEI-LA/miR-124 had reduced NFATc1 expression levels compared with those treated with PHMs/miR-24, RNAiMAX/miR-124, or M-PHMs/miR-124 negative control (M-PHMs/miR-124 NC). M-PHMs/miR-124 NC referred to M-PHMs loaded with negative control miR-124. The results demonstrated that miR-124 delivered by M-PHMs and MTX-PEI-LA had been efficiently transfected into the cells and suppressed the expression of NFATc1. RAW 264.7 cells induced by LPS were then adopted to measure the cytotoxicity of the MTX conjugates through methyl thiazolyl tetrazolium (MTT) test. As shown in **Figure S6**, no cytotoxicity was observed for miR-124 in LPS-induced RAW 264.7 cells. Free MTX, MTX-PEI-LA, M-PHMs, M-PHMs/miR-124 NC, MTX-PEI-LA/miR-124, and M-PHMs/miR-124 (MTX=0.29 µg/mL, miR-124=50 nM) had similar cytotoxicity after 48 h. Surprisingly, MTX-PEI-LA, MTX-PEI-LA/miR-124, and M-PHMs/miR-124 had a little stronger cytotoxicity than free MTX. This might be caused by the enhanced cellular uptake of MTX-PEI-LA compared with free MTX 55. However, RNAiMAX/miR-124 showed the strongest cytotoxicity to the LPS-induced RAW 264.7 cells. The high cytotoxicity was caused by the carrier RNAiMAX. The results suggested that although RNAiMAX had a high transfection efficiency (**Figure 3**), the carrier cytotoxicity cannot be ignored (**Figure S6**). Compared to RNAiMAX, M-PHMs exhibited lower carrier toxicity when complexed with the same amount of miR-124. Although MTX-PEI-LA/miR-124 showed a higher cellular uptake, transfection efficiency, and cytotoxicity compared with M-PHMs/miR-124 *in vitro* (**Figure 3, Figure S5 and Figure S6**), MTX-PEI-LA was still unsuitable for* in vivo* use due to its higher positive charge, BSA precipitation rate and potential instability in the blood (**Figure S3**). Therefore, RNAiMAX and MTX-PEI-LA were not included in the *in vivo* therapeutic studies.

### Hemolytic analysis of M-PHMs/miR-124 and PHMs/miR-124

To investigate the blood biocompatibility of the complexes for systemic delivery *in vivo*, a hemolysis test was carried out. As shown in **Figure S7A-C**, no obvious erythrocyte hemolysis was observed in the M-PHMs/miR-124, PHMs/miR-124, and MTX groups. The hemolytic ratios of MTX, M-PHMs/miR-124, and PHMs/miR-124 were both below 2.5%, suggesting good biocompatibility and safety when administered intravenously (**Figure S7D**). Good biocompatibility may depend largely on the PEG layer in the particles 68. Besides, M-PHMs/miR-124 complexes had a lower hemolytic activity compared with PHMs/miR-124. This may be related to the lower positive charge on the surface of M-PHMs/miR-124 complexes (**Table S2**).

### *In vivo* biodistribution of M-PHMs/miR-124 in inflamed joints

**Figure 5:**
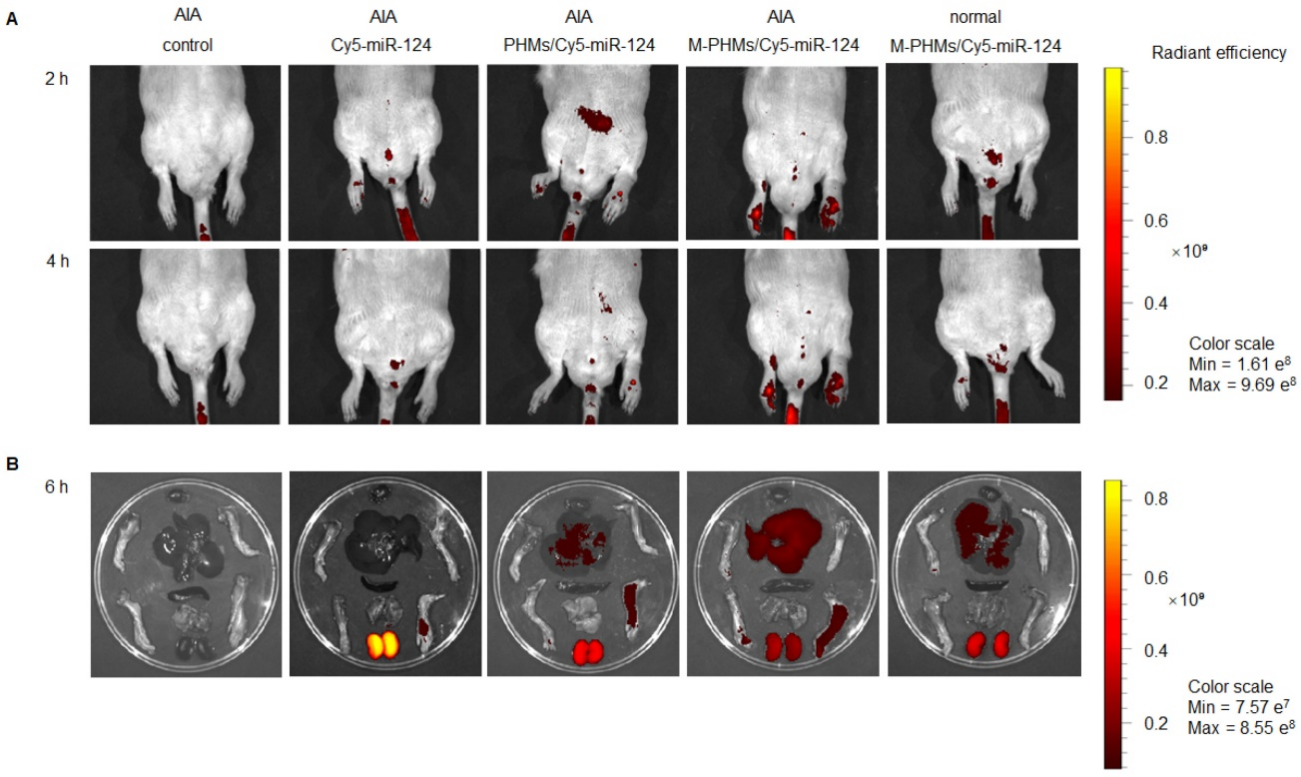
Bio-distribution of M-PHMs/miR-124 *in vivo*. (A) Real-time fluorescence imaging of AIA rat injected with the naked Cy5-miR-124, PHMs/Cy5-miR-124 or M-PHMs/Cy5-miR-124 (Cy5-miR-124=2 nmol) via the tail vein was obtained at 2 and 4 h after administration. (B) Ex vivo imaging of the paws and organs excised from AIA and normal rats was imaged at 6 h after injection. The AIA rat injected with saline and the normal rat injected with M-PHMs/Cy5-miR-124 were set as the blank and negative control, respectively. Images were obtained by IVIS^R^ *In Vivo* Imaging System with an optimized parameter (excitation, 640 nm; emission, 700 nm).

Although the M-PHMs/miR-124 could be efficiently taken up by the activated macrophages with high FR expression *in vitro*, efficient co-delivery of microRNA and MTX to the activated macrophages in inflamed joints was a prerequisite for successful therapy in RA. Thus, the fluorescence intensity of the complexes in AIA rats was investigated and quantified (**Figure 5 and Figure S8**). As shown in** Figure 5**, the naked Cy5-miR-124 was quickly cleared by the kidney* in vivo*. The fluorescence intensity percentage of naked Cy5-miR-124 in the kidney was 81.9%, while the fluorescence intensity percentage in liver and joints is only 10.5% and 5.6% (**Figure S8**). The AIA rat injected with M-PHMs/Cy5-miR-124 complexes exhibited the highest fluorescence intensity in inflammatory joints (**Figure 5**). The percentage of fluorescence intensity at the inflammatory joint site in the M-PHMs/Cy5-miR-124 treatment group was 1.3-fold and 3-fold higher than that of the PHMs/Cy5-miR-124 complex and the naked Cy5-miR-124 (**Figure S8**). Compared with PHMs/Cy5-miR-124 group, M-PHMs/Cy5-miR-124 group showed a significant fluorescence intensity percentage increase in the liver (54.4% vs. 34.8%), while there was a reduction in the kidneys (24.1% vs. 46.7%) (**Figure S8**). The positive charge on the PHMs/Cy5-miR-124 complexes (**Table S2**) may result in the rapid clearance of PHMs/Cy5-miR-124* in vivo*. PHMs/Cy5-miR-124 group was also observed a much higher distribution in the inflamed joints than the naked Cy5-miR-124 group. This may be due to the passive targeting of nanosized particles through enhanced permeability and retention effect (EPR) in inflammation sites as previous studies indicated 29, 71, 72. The studies confirmed that M-PHMs/Cy5-miR-124 could efficiently target and specifically deliver Cy5-miR-124 to the inflamed joints. The previous study has shown that FR-targeted nanocarriers are more likely to accumulate at sites of inflammation than tumors that express high FR 72. Therefore, M-PHMs/Cy5-miR-124 targeting activated macrophages may have a good prospect in RA therapy 73.

### Therapeutic efficacy of M-PHMs/miR-124 in AIA rat model

**Figure 6:**
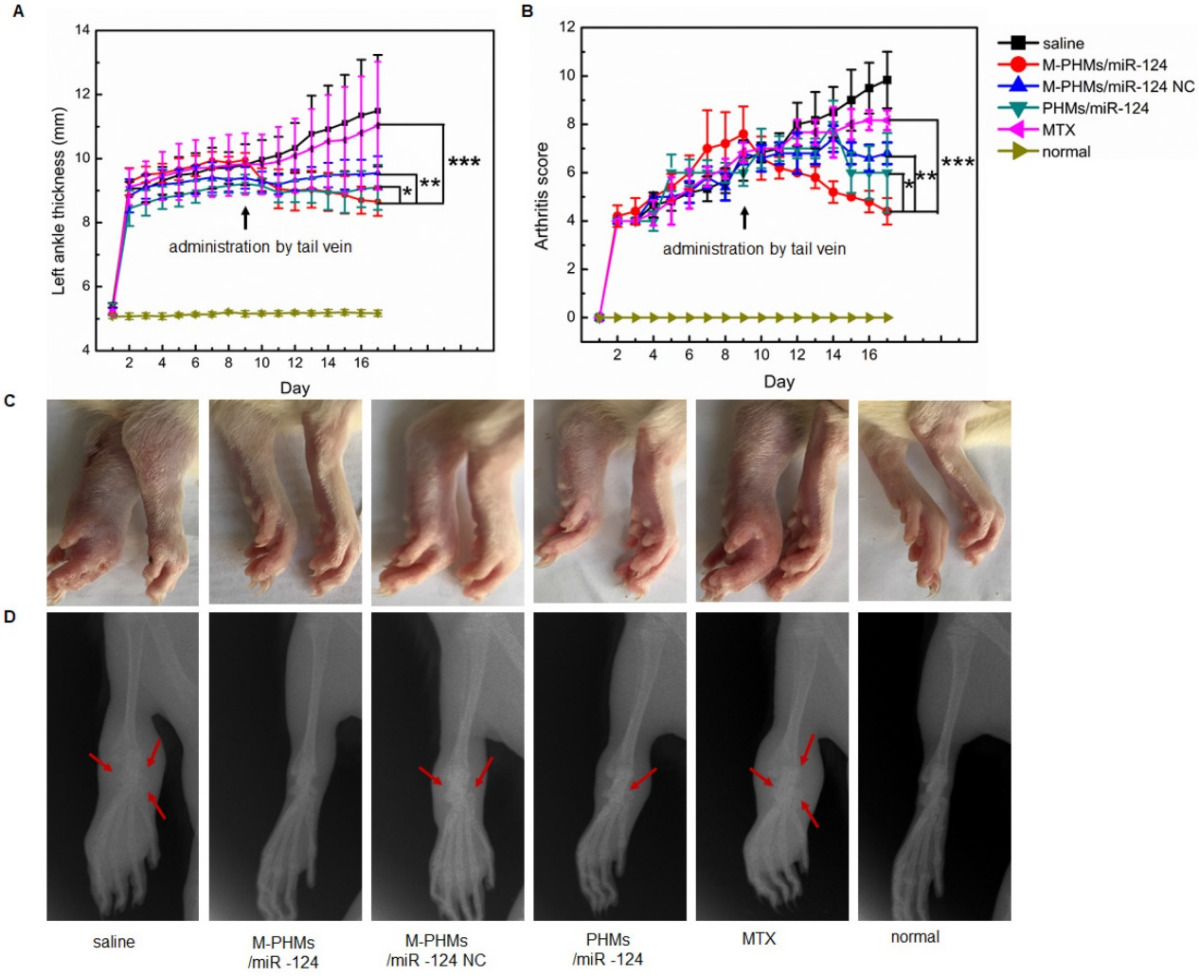
Therapeutic efficacy of M-PHMs/miR-124 *in vivo*. Left ankle thickness (A) and clinical score of AIA rats (B) treated with various drug formulations were obtained (*P < 0.05, **P < 0.01, ***P < 0.001, PHMs/miR-124, PHMs/miR-124 NC, and MTX separately compared with M-PHMs/miR-124). (C) Photographs of hind paws and representative X-ray of the left ankle (D) from AIA rats treated with various drug formulations. Rats without AIA were set as normal group. Values are mean ± SEM (n=6).

Bone changes assessed are more severe in AIA rats than in collagen-induced arthritis models. The hallmark of the AIA model is reliable disease initiation and progression, and it is easy to measure articular inflammation and significant bone destruction 74, 75. Thus, to evaluate the synergy of MTX and miR-124, AIA model was established. Anti-inflammatory effect on AIA rats was evaluated by the measurement of the hind paws thickness, scores of the fore and hind paws, photographs of hind paws (**Figure 6A-C and Figure S9**). Bone changes in joints were assessed by X-ray photograph (**Figure 6D**). As shown in **Figure 6A-B**, left hind paw thickness and arthritis score of saline and free MTX treated animals were gradually increased indicating disease progression, while this progression was decreased in animals treated with M-PHMs/miR-124 NC, PHMs/miR-124 and M-PHMs/miR-124. Animals treated with M-PHMs/miR-124 were found to be a highly significant symptom remission compared with MTX (***P < 0.001), M-PHMs/miR-124 NC (**P < 0.01) and PHMs/miR-124 (* P < 0.05) in paw thickness, swelling, and erythema score. AIA rats treated with M-PHMs/miR-124 NC and PHMs/miR-124 were also found to have reduced paw thickness, swelling and erythema score compared with saline and MTX group. In addition, as shown in** Figure S9**, only the right ankle thickness of AIA rat treated with saline was greatly increased. This indicates that the drug-administered groups can inhibit the disease progression of the contralateral joint. Similar results were also observed from the photographs of hind paws (**Figure 6C**). Compared with the clearly visible of joints by X-ray photograph with uniform and integrity in normal rats and M-PHMs/miR-124 treated groups, physiological saline and free MTX treated animals had a vague shadow, incompleteness of bone structure and an obvious bone loss and damage as the red arrow indicated (**Figure 6D**). The integrity of articular cartilage in the PHMs/miR-124 group was found to be superior to that of the M-PHMs/miR-124 NC, MTX and saline groups as the red arrows indicated. Both M-PHMs/miR-124 and PHMs/miR-124 group had obvious bone protection. This suggested strong bone protection of miR-124 as previous studies reported 27.

As shown in **Figure 5 and Figure S8**, M-PHMs/Cy5-miR124 complexes injected to the AIA rat showed a high fluorescence intensity in the liver compared to other groups. Therefore, histological analysis of liver excised from groups treated with different formulations was investigated to justify whether MTX in M-PHMs/miR-124 can cause hepatotoxicity. As shown in **Figure S10**, there was no obvious hepatotoxicity in the groups treated with saline, M-PHMs/miR-124, M-PHMs/miR-124 NC, PHMs/miR-124, and MTX compared with the normal group. Actually, the dose of the MTX (38 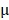
g/kg) via tail vein injection was low and could not cause toxicity even a high accumulation of M-PHMs/miR-124 in the liver. Body weight of the AIA rats was also measured to confirm whether the micelles had systemic toxicity upon the drug administration. The body weight of AIA rats treated with different formulations was found to be steadily increased and there was no significant difference **(Figure S11)**. The formulations had no obvious injection toxicity.

### The serum concentration of pro-inflammatory cytokines in AIA rats

**Figure 7:**
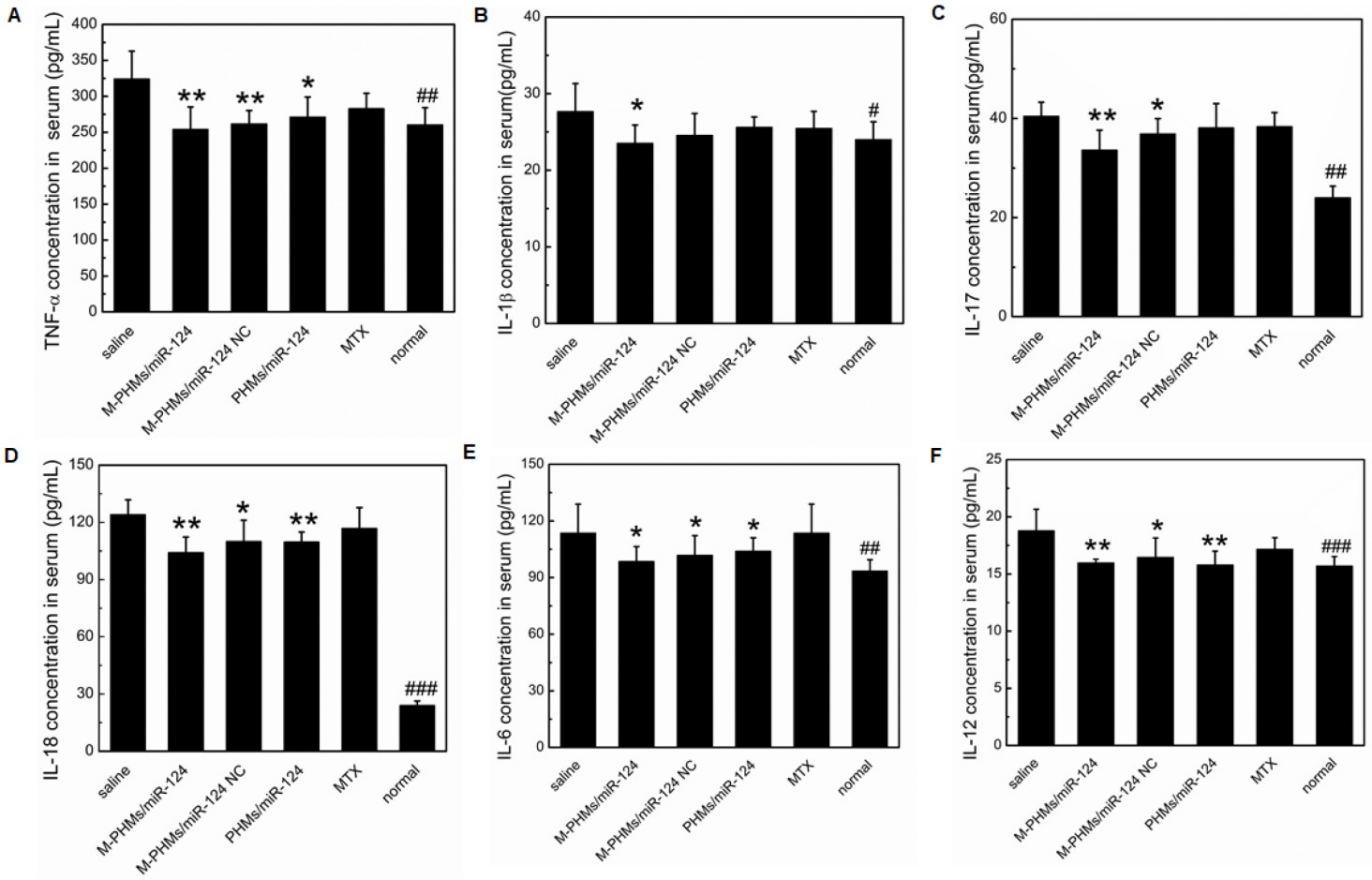
The serum concentration of pro-inflammatory cytokines. Serum levels of the pro-inflammatory cytokines TNF-α (A), IL-1β (B), IL-17 (C), IL-18 (D), IL-6 (E) and IL-12 (F) were measured in AIA rats treated with different formulations (n=6, mean ± SEM, *P < 0.05, **P < 0.01, ***P < 0.001, M-PHMs/miR-124, M-PHMs/miR-124 NC, PHMs/miR-124, and MTX treated AIA rats separately compared with AIA rats treated with saline group, ^#^P < 0.05, ^##^P < 0.01, ^###^P < 0.001, normal rat group compared with AIA rats treated with saline).

Cytokines play a central role in the progression of arthritic diseases 2. Enhanced levels of pro-inflammatory crucial cytokines such as TNF-α, IL-1β, IL-17, IL-18, IL-6, IL-12 often result in chronic inflammatory responses and destruction of joints 76, 77. Therefore, the serum expression levels of crucial pro-inflammatory cytokines in AIA rats treated with different formulations were determined for further evaluation of the progression of the arthritis disease. As shown in **Figure 7**, TNF-α, IL-1β, IL-17, IL-18, IL-6, IL-12 expression levels were significantly inhibited in the serum of M-PHMs/miR-124 treated group. Compared with M-PHMs/miR-124 NC and PHMs/miR-124 groups, M-PHMs/miR-124 treated group had the least concentration of TNF-α (**P < 0.01, **Figure 7A**), IL-1β (*P < 0.05, **Figure 7B**) and IL-17 (**P < 0.01, **Figure 7C**). This indicated a synergy of MTX and miR-124 in reducing the expression of TNF-α, IL-1β, and IL-17. A reduced serum levels of TNF-α (**P < 0.01), IL-17 (*P < 0.05), IL-18 (*P < 0.05), IL-6 (*P < 0.05, **Figure 7E**), IL-12 (*P < 0.05) were found in M-PHMs/miR-124 NC group (MTX conjugates alone). AIA rats treated with PHMs/miR-124 (miR-124 alone) were also found to have a reduced TNF-α (*P < 0.05), IL-6 (*P < 0.05), IL-18 (**P < 0.01), IL-12 (**P < 0.01). Compared with PHMs/miR-124 treatment group, M-PHMs/miR-124 NC group showed a stronger inhibition effect of TNF-α, IL-1β, IL-17, and IL-6 expression which were often measured to confirm the efficacy of MTX as previous studies indicated 35, 78. While in AIA rats treated with PHMs/miR-124, the serum levels of IL-18 (**Figure 7D**) and IL-12 (**Figure 7F**) was lower than M-PHMs/miR-124 NC group. There may be a close relation between miR-124, IL-12, and IL-18. In conclusion, the combination of MTX and miR-124 suggested a synergistic inhibition of cytokines expression.

### Histopathologic analysis of joints in AIA rats

**Figure 8:**
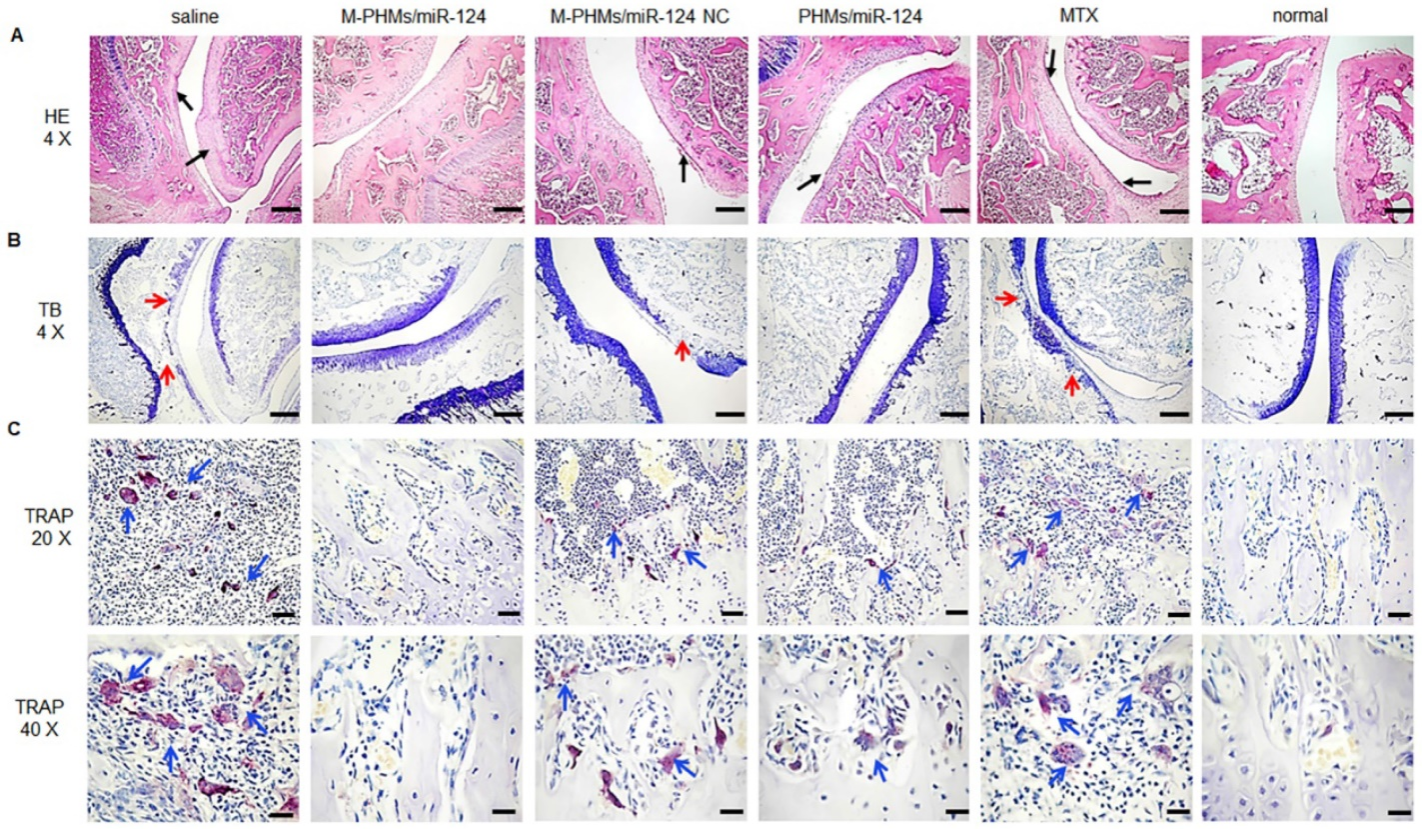
Histopathological examination of AIA rat joints. Hematoxylin-eosin (HE), toluidine blue (TB) and tartrate-resistant acid phosphatase (TRAP) staining analyses were performed on joints section from AIA rats treated with saline, M-PHMs/miR-124, M-PHMs/miR-124 NC, PHMs/miR-124 and MTX. Histopathological examination of normal rat group was set as a blank control. (A) HE staining was applied to study synovial hyperplasia. Black arrows indicated severe synovial hyperplasia. (B) TB staining of joints was conducted to analyze the integrity of articular cartilage. The red arrows indicated severe destruction and incompleteness of articular cartilage. (C) TRAP staining was used to study the number of osteoclasts. The blue arrows referred to the clusters of osteoclasts. The scale bar in HE (4 X, A) and TB (4 X, B) was 500 µm. The scale bars in TRAP (20 X and 40 X) were 100 µm and 50 µm, respectively.

The histopathologic analysis was applied to observe the pathological changes in joints of AIA rats treated with different formulations (**Figure 8**). The ankle joint sections stained with hematoxylin-eosin (HE), toluidine blue (TB), tartrate-resistant acid phosphatase (TRAP) were conducted to investigate synovial hyperplasia, cartilage and bone erosion, and osteoclast generation in animals treated with different formulations. Black arrows indicated severe synovial hyperplasia and cellular infiltrates (**Figure 8A**). The red arrows indicated severe destruction and incompleteness of articular cartilage (**Figure 8B**). The blue arrows referred to the generation of osteoclasts (**Figure 8C**). Stained ankle joints of AIA rats treated with saline and free MTX revealed the most severe synovial hyperplasia, degradation and destruction of articular cartilage tissue, and contained the most osteoclast clusters. Synovial hyperplasia was somewhat less extensive in animal groups treated with M-PHMs/miR-124 NC and PHMs/miR-124, as shown in **Figure 8A**. Animals treated with M-PHMs/miR-124 complexes showed the mildest histopathology, with clear and smooth cartilage, fewer and lighter synovial hyperplasia and less infiltration by inflammatory cells compared with other groups in **Figure 8A**. Similar results were obtained by analyzing ankle joint sections stained with TB, which indicated the degree of cartilage destruction (**Figure 8B**). The osteoblasts could be colored to blue by TB. Animals treated with M-PHMs/miR-124 complexes had cartilage as intact as normal rats. Compared to the M-PHMs/miR-124 NC, cartilage was less damaged in animals treated with PHMs/miR-124. The results were consistent with the results of** Figure 6D** and further demonstrated the cartilage protection of miR-124. However, M-PHMs/miR-124 NC also showed weak cartilage protection. Due to the pro-inflammatory cytokines often resulted in joint and cartilage destruction, the weak cartilage protection may be caused by the reduced pro-inflammatory cytokines of M-PHMs/miR-124 NC (**Figure 7**).

miR-124 was previously reported to inhibit the progression of AIA rats by reducing the osteoclast differentiation 25, 27, 28. Thus, TRAP staining was further performed to investigate the number of osteoclasts in joints of AIA rats. Purple stain area referred to the osteoclasts clusters as blue arrows indicated (**Figure 8C**) and the TRAP (+) cells number and TRAP (+) area were further quantified by using Image pro-plus 6.0 (**Figure S12**). As shown in **Figure 8C and Figure S12**, saline and free MTX group had a large number of osteoclasts (13.6 folds and 10.8 folds than normal group per cm^3^) generation and much larger purple stain area (24.4 folds and 16.1 folds purple area than normal group). The increased osteoclasts may be a major cause for severe cartilage destruction of the two groups. M-PHMs/miR-124 complexes treated AIA rats had the least of TRAP (+) cells number (***P < 0.001) and the smallest TRAP (+) area (**P < 0.01) compared with M-PHMs/miR-124 NC, PHMs/miR-124, MTX and saline groups. The TRAP (+) cells number of M-PHMs/miR-124 NC, PHMs/miR-124, MTX and saline groups was 3.87 folds, 3.00 folds, 4.12 folds, and 5.12 folds that of M-PHMs/miR-124. The TRAP (+) area in M-PHMs/miR-124 NC, PHMs/miR-124, MTX and saline groups was 3.89 folds, 2.90 folds, 7.65 folds, and 11.57 folds that of M-PHMs/miR-124. The results of TRAP staining further exhibited that the miR-124 in M-PHMs/miR-124 was most efficiently delivered to the macrophages in inflamed joint, reduced the number of osteoclasts, and ultimately prevented the bone destruction.

Despite the long clinical history of MTX in RA, the exact mechanism of low-dose MTX anti-inflammatory action is still not fully understood. The enhancement of adenosine signaling has been strongly supported by animal model studies and some of which even have been confirmed in humans 16. As shown previously, MTX or MTX conjugates therapy can be assessed by measuring the thickness of the swollen joints, clinic score, and proinflammatory cytokines levels and histopathological analysis of joints 34, 35, 47. miR-124 has a direct bone protective effect in the context of RA by suppressing the differentiation of human osteoclasts and has anti-inflammatory activity in RA by inhibiting proinflammatory cytokines levels 26, 27. To assess the bone protection of M-PHMs/miR-124, we performed an X-ray imaging, TB and TRAP staining analysis of joints section in AIA rats. Therefore, from the results of **Figure 6**, **Figure 7**, **Figure 8 and Figure S12**, it could be concluded that M-PHMs/miR-124 achieved a combination therapy of MTX and miR-124 in AIA rats.

Compared with free MTX, a mild anti-inflammatory effect was observed in AIA rats treated with M-PHMs/miR-124 NC (MTX conjugates) (**Figure 6, Figure 7, Figure 8 and Figure S12**). This may be due to the good stability and targeting efficiency of MTX conjugates, which finally improved the amount of MTX conjugates in the targeted macrophages 42, 46. Since MTX was conjugated to the PEI-LA with a stable amide bond, as demonstrated in the previous study, the MTX conjugates may work primarily in two ways: 1) MTX conjugates directly bind to the target protein and obtain the therapeutic effects 53, 79, 80; 2) MTX conjugates may release MTX through cytoplasmic protease-mediated peptide bond cleavage due to the presence of many types and functions of proteases in cells or lysosome 81-83. Therefore, the release of free MTX may not be necessary to achieve the therapeutic efficacy of MTX conjugates. The MTX-PEI-LA conjugates in M-PHMs may be an active agent of RA treatment.

A moderate therapeutic efficiency was also observed by PHMs/miR-124 treated group. This might be due to the EPR effect of PHMs that promotes the accumulation of miR-124 in the joint and might also be associated with the therapeutic effects of free miR-124. However, as shown in **Figure 5** and **Figure S6**, compared with free miR-124, PHMs/miR-124 group had a higher accumulation of miR-124 (2.3 folds than free miR-124) in joint and much slower clearance rate. The greater miR-124 accumulation in joint may contribute to a good therapeutic effect of PHMs/miR-124. This may be explained by the passive targeting of the PHMs/miR-124 through the EPR effect of nanosized micelles 29, 84, 85.

Above all, based on the results of joint thickness, clinical score, X-ray photograph, serum concentration of pro-inflammatory cytokines and histological analysis of joints in vivo (**Figure 6, Figure 7 and Figure 8**), we concluded that a synergy effect of M-PHMs/miR-124 may mainly depend on the MTX conjugates and miR-124 anti-inflammatory effect and miR-124 bone protection. The results implied that the M-PHMs/miR-124 complexes exhibited a greater effect compared with M-PHMs/miR-124 control and PHMs/miR-124. This might be due to the specific and efficient delivery of miR-124 and MTX conjugates to the activated macrophages in arthritis sites. The efficient therapy effect especially a direct bone protective effect *in vivo* may be explained by the MTX mediated folate receptor targeting and enhanced miR-124 delivery, resulting in the reduction of the osteoclasts generation and suppression of cartilage destruction. However, the specific and detailed therapy mechanism and the synergistic effect of co-delivery of MTX and miR-124 still require further investigation.

## Conclusion

In this work, two polymer components, MTX-PEI-LA, and mPEG-LA were synthesized and used to prepare the dual functional M-PHMs with miR-124 encapsulation by a simplified, tunable and smart approach. The M-PHMs/miR-124 complexes exhibited a strong FRβ targeting ability on the activated macrophages, were internalized by FR-mediated endocytosis, and successfully escaped from the endosome, released miR-124 into the cytoplasm and then down-regulated the expression of NFATc1. Biodistribution and therapeutic activity in an AIA rat model demonstrated that M-PHMs/miR-124 could specifically target the inflamed joints and achieve an enhanced synergistic efficacy of MTX and miR-124 *in vivo*. In addition, we presented a new rational design for drug co-delivery. The bifunctional and efficient drug delivery system with a simple design could be applied to other disease therapy by changing the payload.

## Supplementary Material

Supplementary experimental section, FT-IR spectra of the synthesized amphiphilic polymer, the wavelength scan of MTX-PEI-LA, size and zeta potential of M-PHMs with different ratios of MTX-PEI-LA, size and zeta potential of M-PHMs/miR-124, BSA adsorption assay, miR-124 release in M-PHMs/miR-124, Inhibition of NFATc1 expression, cell viability, hemolysis analysis, fluorescence intensity percentage in dissected organs and joints, right ankle thickness, histological analysis of the liver, body weight of AIA rats, quantitative analysis of TRAP (+) cells number and TRAP (+) area.

## Abbreviations

RA: rheumatoid arthritis
DMARDs: disease-modifying antirheumatic drugs
MTX: methotrexate
miR-124: microRNA-124
AIA: adjuvant-induced arthritis
folate receptor beta FRβ: folate receptor beta
SR: scavenger receptor
FA: folate acid
G5: generation 5 dendrimer
FRα: folate receptor aerfa
PEI: polyethyleneimine
DOX: doxorubicin
MTX-PEI-LA: MTX linked to polyethyleneimine with linoleic acid conjugated
M-PHMs: MTX-conjugated polymer hybrid micelles
mPEG-LA: linoleic acid-modified methyl polyethylene glycol
FT-IR: fourier-transform infrared
LC: linoleyl chloride
CDCl_3_: deuterated chloroform
DMSO-d_6_: deuterated dimethyl sulfoxide
D_2_O: deuterated water
PDI: polydispersity index
BSA: bovine serum albumin
PHMs: polymer hybrid micelles
FESEM: field emission scanning electron microscopy
PEG: polyethylene glycol
CMC: critical micelle concentration
N/P: nitrogen to phosphate ratio
PBS: phosphate buffer solution
LPS: lipopolysaccharide
NFATc1: nuclear factor of activated T cells cytoplasmic 1
RANKL: receptor activator of nuclear factor-κB (NF-κB) ligand
M-PHMs/miR-124 NC: M-PHMs/miR-124 negative control
MTT: methyl thiazolyl tetrazolium
EPR: enhanced permeability and retention effect
H&E: hematoxylin and eosin
TB: toluidine blue
TRAP: tartrate-resistant acid phosphatase
