# Supplementary material for: Hybrid micelles containing methotrexate-conjugated polymer and co-loaded with microRNA-124 for rheumatoid arthritis therapy
Source: Theranostics. 2019 Jul 9;9(18):5282–97. doi: 10.7150/thno.32268 (PMC6691571; doi:10.7150/thno.32268)
Supplement: Supplementary file 1 — Supplementary experimental section, FT-IR spectra of the synthesized amphiphilic polymer, the wavelength scan of MTX-PEI-LA, size and zeta potential of M-PHMs with different ratios of MTX-PEI-LA, size and zeta potential of M-PHMs/miR-124, BSA adsorption assay, miR-124 release in M-PHMs/miR-124, Inhibition of NFATc1 expression, cell viability, hemolysis analysis, fluorescence intensity percentage in dissected organs and joints, right ankle thickness, histological analysis of the liver, body weight of AIA rats, quantitative analysis of TRAP (+) cells number and TRAP (+) area. [file thnov09p5282s1.pdf]

Hybrid micelles containing methotrexate-conjugated polymer and co-loaded with microRNA-124 for rheumatoid arthritis therapy

Fei Hao [1], Robert J. Lee[1,2], Lihuang Zhong[1], Shiyan Dong[1], Chunmiao Yang[1], Lirong Teng[1], Qingfan Meng[1], Jiahui Lu[1], Jing Xie[1], Lesheng Teng[1*]

[1] *School of Life Sciences, Jilin University, No.2699, Qianjin Street, Changchun130012, P.R. China;*

[2] *College of Pharmacy, The Ohio State University, Columbus, 500 W 12th Ave, Columbus, OH 43210, USA;*

Corresponding author：Lesheng Teng, Ph.D., Professor, School of Life Sciences, Jilin University, No.2699, Qianjin Street, Changchun, China, 130012, Tel: (86) 13844181693; Email:tenglesheng@jlu.edu.cn.

Experimental Section

Materials

Branched polyethylenimine（PEI, 25 kDa）and folic acid (FA) were purchased from Sigma-Aldrich (St. Louis, MO, USA). Methotrexate (MTX) and methyl thiazolyl tetrazolium (MTT) were purchased from Shanghai Yuanye Biological Technology Co., Ltd. (Shanghai, China). mPEG-NH_2 (2000 Da) was purchased from Yarebio (Shanghai, China). Linolyl chloride (LC) was obtained from Tokyo Chemical Industry Co., Ltd. (Shanghai, China). Dulbecco's modified Eagle's medium (DMEM) was purchased from Gibco (SuZhou, China). Lipopolysaccharide (LPS) was purchased from Sigma–Aldrich (St. Louis, MO, USA). mmu-miR-124-3p mimic (5'-UAAGGCACGCGGUGAAUGCC3', 3'-AUUCCGUGCGCCACUUACGG5'), mmu-miR-124-3p mimic negative control #22 (5'-UUUGUACUACACAAAAGUACUG-3', 3'-AAACAUGAUGUGUUUUCAUGAC-5'), fluorescence-labeled miR-124 that had Cy3, FAM or Cy5 conjugated to the 5′ end was synthesized by Ribo Biochemistry (Guangzhou, China). RAW 264.7 cells were obtained from Wuhan Procell Biological Technology Co., Ltd. (Wuhan, China). 4′,6-Diamidino-2-phenylindole (DAPI) and Lyso Tracker™Red DND-99 were purchased from Invitrogen Co. (Carlsbad, CA, USA). ELISA kits for TNF-α, IL-6, IL-1β, IL-17, IL-18, and IL-12 were obtained from Shanghai YuanYe Biological Technology Co., Ltd. (Shanghai, China). Complete Freund's adjuvant (CFA) was obtained from Chondrex (Redmond, WA, USA). All other chemicals used were commercially purchased at analytical grade.

Synthesis and characterization of MTX-PEI-LA and mPEG-LA

Synthesis of MTX-PEI-LA was carried out in two steps. First, PEI-LA was synthesized using a previously reported method [1, 2]. Briefly, linolenic chloride (LC) (15 mg) dissolved in anhydrous dichloromethane (DCM) was slowly added to a solution of PEI (105 mg) in 8.5 mL anhydrous DCM containing 500 µL anhydrous triethylamine (Et_3N). Diethyl ether was used to precipitate the PEI-LA product. The product was then washed 3 times with diethyl ether. PEI-LA was obtained by removing the organic solvent on a rotary evaporator and under vacuum for 2 h. MTX (35.9 mg) dissolved in 9 mL dimethylformamide (DMF) was firstly activated for 4 h with 1-hydroxybenzotriazole (HOBT, 198 µmol), O-benzotriazole-N, N, N', N'-tetramethyl-uronium-hexa-fluorophosphate (HBTU, 198 µmol), and N, N-diisopropylethylamine (DIEA, 396 µmol). The PEI-LA dissolved in 9 mL anhydrous methanol was then slowly added dropwise into the MTX solution. The mixture was incubated with gentle stirring at room temperature for 24 h under nitrogen atmosphere. The resulting reaction mixture was then placed in a dialysis bag with a molecular weight cutoff (MWCO) of 8,000 to 14,000 Da and dialyzed against deionized water, with changing of dialysate every 4 h. After 48 h, MTX-PEI-LA was placed in a freeze dryer（Christ epsilon 2-6D LSC, Osterode, Germany）to remove water. mPEG-LA was obtained by a similar method to PEI-LA. Briefly, 16.5 mg of LC dissolved in 6 mL anhydrous DCM was added dropwise to 102 mg of mPEG-NH_2 dissolved in anhydrous DCM containing 500 µL Et_3N with moderately stirring in a nitrogen environment at room temperature. After 12 h, the final product was obtained and dried under vacuum.

The chemical structures of mPEG-2000 Da-NH_2, PEI-25 kDa, LC, MTX, mPEG-LA, PEI-LA, and MTX-PEI-LA were confirmed by ^{1}H NMR on a 500 MHz spectrometer and FT-IR (VERTEX 80V) from Bruker (Fällanden, Switzerland). mPEG-2000 Da-NH2, PEI-25 kDa, LC, PEI-LA, and mPEG-LA were analyzed using deuterated chloroform ($CDCl_3$) as the solvent. MTX was dissolved in deuterated dimethyl sulfoxide (DMSO-d6), while MTX-PEI-LA was in deuterated water (D_2O). mPEG-2000 Da-NH2, PEI-25 kDa, LC, MTX, mPEG-LA, PEI-LA, and MTX-PEI-LA were mixed with potassium bromide (KBr) (Tianjin Guangfu Fine Chemical Research Institute, Tianjin, China), ground into a powder and dried before the samples were analyzed. Spectra of MTX, PEI, LC, PEI-LA, MTX-PEI-LA were then obtained on a Shimadzu UV-2401PC UV-VIS spectrophotometer (Tokyo, Japan). The MTX content in the MTX-PEI-LA was determined by measuring the absorbance of the MTX-PEI-LA. The MTX content was calculated based on a calibration curve which was constructed from a series of standard MTX solutions of known concentrations. The drug loading efficiency was calculated as follows:

Drug loading efficiency (%)= weight of MTX conjugated to PEI-LA / total weight of MTX-PEI-LA

Preparation of MTX-conjugated polymeric hybrid micelles (M-PHMs) with different weight

percentages of MTX-PEI-LA

M-PHMs were prepared by a thin film evaporation method. Briefly, MTX-PEI-LA and mPEG-LA dissolved in chloroform were combined at different mass ratios and sonicated for 3 min at room temperature. Then the organic solvent of the mixture solution was removed at 37 ℃ on a rotary evaporator and further vacuumed for 2 h to remove residual organic solvents and to obtain a thin film on the flask. To prepare M-PHMs, diethyl pyro carbonate-treated (DEPC) water was added into the flask, which was sonicated for 2 min. Particle size, zeta potential, and polydispersity index (PDI) of M-PHMs with different weight percentages of MTX-PEI-LA (0, 2, 5, 10, 20, 50, 80, 100%) were measured by dynamic light scattering (DLS) on a Zeta-sizer Nano ZS90 from Malvern Instruments (Malvern, UK) at 25℃.

The colloidal stability of the M-PHMs in serum with different weight percentages of

MTX-PEI-LA

The colloidal stability of the M-PHMs in serum was performed by bovine serum albumin (BSA) precipitation assay, as described in a previous study [3]. Briefly, 500 µL of M-PHMs with different mass ratios was incubated with 500 µL of BSA solution (10 mg/mL) for 4 h at 37 ℃. Then the mixture was centrifuged at 5000 rpm for 20 min and the supernatant was carefully collected. The concentration of BSA in the supernatant was measured using a BCA protein assay kit from Thermo Scientific (Rockford, IL., USA). BSA precipitation ratio was calculated using the following equation:

BSA precipitation ratio (%)=$(m-m_0) / m \times 100\%$

"m" represented the initial total amount of BSA. "m_0" was the BSA amount in the supernatant after centrifugation.

The characterization of the M-PHMs with an optimized percentage of MTX-PEI-LA

The optimal mass percentage of MTX-PEI-LA in M-PHMs was obtained based on the values of particle size, zeta potential, and colloidal stability in serum. In order to better characterize the M-PHMs properties, the polymer hybrid micelles (PHMs) without MTX conjugation were also prepared with the same optimized mass ratio of PEI-LA and mPEG-LA. The morphologies of MTX, MTX-PEI-LA, PHMs, and M-PHMs with the optimized percentage of MTX-PEI-LA were then imaged by field emission scanning electron microscopy (FESEM, JSM-6700F, JEOL, Tokyo, Japan) with an accelerating voltage of 300 kV on silicon wafers. Next, the critical micelle concentration (CMC) of the optimal M-PHMs and PHMs was further determined by a classical method based on conductivity.

Gel Retardation Assay

To investigated the ability of M-PHMs or PHMs to complex miR-124, gel retardation assays were performed using agarose gel electrophoresis. The micelles (including M-PHMs and PHMs) and miR-124 solutions were first diluted to a series of concentrations to prepare micelles/miR-124 complexes at different nitrogen to phosphate ratios (N/P). The desired amount of miR-124 solution was then mixed with an equal volume of micelles solution by gentle pipetting. Prior to use, the complexes were incubated for 10 min at room temperature. Then, 10 μL of the micelles/miR-124 complexes with different N/P were mixed with 2 μL of 6 × loading dye and loaded into 2% agarose gel containing ethidium bromide. Electrophoresis was set up at 80 V and run for 20 min in a Tris-acetate-EDTA buffer. Free miR-124 in the complexes could be detected as a band on the gel with a UV transilluminator (Analytik Jena US LLC., Upland, CA, USA).

Characterization and miR-124 release profile of M-PHMs/miR-124 complexes

Particle size, zeta potential and PDI of the M-PHMs/miR-124 complexes and PHMs/miR-124 complexes formed at an optimal N/P were determined by Zeta-sizer Nano ZS90. The release of miR-124 loaded in the M-PHMs/miR-124 complexes was further investigated in phosphate buffer saline (PBS) (pH=7.4). The M-PHMs/FAM-miR-124 complexes were prepared and placed into a dialysis bag with a molecular weight cut-off (MW) of 100 kDa. FAM-miR-124 released at regular intervals was obtained and measured, as described previously [4]. The fluorescence intensity of FAM-miR-124 was determined by a microplate reader (Bio Tek SYNERGY4, Winooski, VT, USA) (excitation, 485 nm; emission, 535 nm). The concentration of the released FAM-miR-124 was calculated based on a standard curve of FAM-miR-124 with known concentration.

Cell culture

RAW 264.7 cells were used to evaluate folate receptor (FR) mediated uptake of the M-PHMs. Cells were cultured in DMEM containing 10% fetal bovine serum (FBS) and 1% penicillin-streptomycin at 37 ℃ in a humidified atmosphere of 5% CO_2. To obtain activated macrophages with high expression of folate receptor beta (FRβ), RAW 264.7 cells were incubated with LPS (1 μg/mL) for 48 h.

FR-mediated cellular uptake of the M-PHMs/Cy3-miR-124 complexes

Flow cytometry was first used to identify cellular uptake of M-PHMs/miR-124 complexes in LPS-induced {LPS (+)} or non-induced {LPS (–)} RAW 264.7 cells. Briefly, activated or non-activated cells were seeded in 12-well cell culture plates at a concentration of 1×10^5/well for 12 h. Additionally, the cells were pre-incubated with free FA with varying concentrations (1 mM, 0.1 mM and 0.01 mM) for 1 h to investigate the competitive inhibition of FA to the uptake of the M-PHMs/Cy3-miR-124 complexes. After 4 h of incubation with MTX-PEI-LA/Cy3-miR-124, RNAiMAX/Cy3-miR-124, M-PHMs/Cy3-miR-124, Cy3-miR-124, or PHMs/Cy3-miR-124 (equivalent concentration of Cy3-miR-124 100 nM), cells were trypsinized, harvested by centrifugation, washed with cold PBS and resuspended with 4% (w/v) formaldehyde solution (Beijing Dingguo Changsheng Biotechnology Co., Ltd., Beijing, China). The fluorescent intensity of the cells was measured on a Beckman Coulter EPICS XL flow cytometer (Brea, CA, USA). Then the cellular uptake was further visualized on a confocal laser scanning microscopy (CLSM). Activated and non-activated macrophages cells were collected, counted, and then seeded at the bottom of the glass flask for 12 h. The medium was replaced with fresh opti-MEM and pre-incubated with free FA (1 mM) for 1 h. Then MTX-PEI-LA/Cy3-miR-124, RNAiMAX/Cy3-miR-124, M-PHMs/Cy3-miR-124, Cy3-miR-124, and PHMs/Cy3-miR-124 (equivalent concentration of Cy3-miR-124 100 nM) were incubated with cells at 37℃. After 4 h of incubation, the cells were washed gently 3 times with PBS (0.01 M, pH=7.4) and then fixed with 4% (w/v) formaldehyde for 10 min at room temperature. The cells were then washed three times with PBS. Subsequently, nuclei were stained with DAPI for 10 min and then the cells were washed with PBS to remove the residual dye. The cellular uptake of different formulations in cells was observed using LSM710 microscope from Carl Zeiss (Oberkochen, Germany).

Endosome escape of M-PHMs/FAM-miR-124 complexes in activated macrophages

Activated macrophages were seeded on glass bottom cell culture dishes and cultured in 1×10^5 cells per dish for 12 h. The medium was replaced with fresh opti-MEM and cells were incubated with M-PHMs/FAM-miR-124 complexes for 1, 2, 4, and 6 h. After being washing with PBS, cells were incubated with Lyso Tracker™ Red DND-99 for 30 min. Then, the supernatant was removed. The cells were gently washed, fixed and then stained with DAPI. After washing away the residual dye, the internalization and endosome escape of miR-124 was observed on CLSM.

Western blotting

The nuclear factor of activated T cells cytoplasmic 1 (NFATc1) expression level was investigated by western blotting. RAW 264.7 cells were seeded in 6-well cell culture plates at a concentration of 1×10^5 cells per well. After 24 h, RAW 264.7 cells were separately transfected with miR-124, MTX-PEI-LA/miR-124, RNAiMAX/miR-124, M-PHMs, M-PHMs/miR-124 negative control (M-PHMs/miR-124 NC), PHMs/miR-124, and M-PHMs/miR-124, and the cells were stimulated with 50 ng/mL receptor activator of the nuclear factor-κB (NF-κB) ligand (RANKL) for 48 h. M-PHMs/miR-124 NC referred to

M-PHMs loaded with negative control miR-124. Then the cells of different groups were collected, lysed in radioimmunoprecipitation assay buffer (RIPA) at 4℃ for 10 min. Protein fractions were then collected by centrifugation and quantified by bicinchoninic acid (BCA) Protein Assay Kit (Thermo Scientific, Rockford, IL, USA). Protein samples were subjected to polyacrylamide gel electrophoresis containing 10% sodium dodecyl sulfate (SDS-PAGE) and run for about 90 min. The protein was then transferred to a polyvinylidene fluoride (PVDF) membrane (Merck Millipore, Billerica, MA) for 2 h under 100 V. The membrane was blocked with 5% BSA solution (w/v) for 4 h and incubated with NFATc1 rabbit mAb (Cell Signaling Technology Inc, Danvers, MA, USA) and GAPDH (Elabscience, Wuhan, China) antibodies overnight, respectively. Horseradish peroxidase (HPR)-conjugated secondary antibody (Elabscience, Wuhan, China) was then incubated with the membranes at 4 ℃ for 4 h and the corresponding protein expression was visualized by Bio Spectrum 600 (Analytik Jena US LLC., Upland, CA, USA) using an electrochemiluminescence (ECL) detection kit (Merck Millipore, Billerica, MA, USA).

Cytotoxicity of M-PHMs/miR-124 *in vitro*

RAW 264.7 cells were seeded in 96-well microtiter plates (8000 cells per well) and stimulated with LPS (1 μg/mL) for 48 h. The cells were treated with miR-124, MTX, MTX and miR-124, MTX-PEI-LA, M-PHMs, M-PHMs/miR-124 NC, PHMs/miR-124, MTX-PEI-LA/miR-124, RNAiMAX/miR-124, and M-PHMs/miR-124 (MTX=0.29 μg/mL, miR-124=50 nM). After 48 h, the cell viability was obtained by MTT assay. The wells treated with PBS were set as the control group. The cell viability was obtained by calculating the percentage viability of the cells that were treated with different formulations compared to the control group, the viability of which was defined to be 100%.

Hemolytic analysis

For systemic delivery, analysis of hemolysis was imperative. Hemolytic analysis of the micelles/miR-124 complexes and MTX was conducted according to a previously published method [5]. Briefly, fresh blood samples were collected from the orbital sinus in sterilized centrifuge tubes. Subsequently, red blood cells (RBCs) were obtained by centrifugation at 1500 rpm for 15 min at 25 ℃. RBCs were washed with saline 3 times and then diluted to a 2% standard dispersion (v/v). Then, 200 μL of various concentrations of MTX, M-PHMs/miR-124, and PHMs/miR-124 were added to 1 mL of 2% standard dispersion, mixed gently, and then incubated in a water bath at 37℃. Saline and 1% Triton X-100 were set up as the negative control and positive control, respectively. After 3 h, all tubes were centrifuged at 1500 rpm for 15 min and the supernatants were collected. An aliquot of the supernatant (100 μL) was added to a 96-well plate and the free hemoglobin released was measured at 540 nm. The hemolysis rate was calculated using the following equation:

Hemolysis rate (%)=$(A_{sample} - A_{negative})/(A_{positive} - A_{negative})$

"A_{sample}" represented the absorbance of samples group. "$A_{negative}$" represented the absorbance of the saline group. "$A_{positive}$" represented the absorbance of 1% Triton X-100 group.

Establishment of a rat model of RA

Male Sprague-Dawley (SD) rats (160-180 g) were obtained from the Experimental Animal Center of Jilin University (Changchun, China) and kept in gopher food and drinking water and kept at a constant temperature. Three rats were housed in each cage. The animal experimental protocol was approved by the Experimental Animal Ethics Committee of the School of Life Sciences, Jilin University. The protocol number is 201704006.

Rat adjuvant arthritis is an experimental model of polyarthritis that has been widely used in preclinical testing of many anti-arthritic drugs [6]. The hallmarks of onset and progression of this animal model are polyarticular inflammation and significant bone destruction [7]. To study the inflammatory effect and bone protection of MTX and miR-124, we chose an adjuvant-induced arthritis (AIA) rat model. Male rats are commonly used in the study of adjuvant arthritis. To induce AIA rat model, 0.05 mL of CFA containing 10 mg/mL of heat-killed M. tuberculosis was subcutaneously injected into a rear paw of left footpad of each rat (day 0), after the rats were acclimated to the new environment for one week. After injection into the footpad, the acute inflammatory response in the local area and the immune response in the contralateral paw after approximately 9 days (onset of disease) can be studied. Hind paw swelling, paw thickness, and clinical score are monitored from day 0 to 18. The body weight of the rat was also measured every day. From the onset of disease (Day 9), drugs were administered by tail vein every two days. Then, 18 days later, photographs of hind paws morphology and radiography by X-ray imaging were obtained. Blood samples were collected from the rat's orbit for biochemical analysis of cytokines. Finally, the paws and liver were dissected and fixed by 4% paraformaldehyde (w/v) for histopathology examination to study the beneficial effects of the carrier.

Accumulation of M-PHMs/Cy5-miR-124 complexes at inflamed joints

Rats with AIA were injected with Cy5-miR-124 (2 nmol) via the tail vein, either as free Cy5-miR-124 or packaged in M-PHMs or PHMs. At 2 and 4 h after administration, the biodistribution of M-PHMs/Cy5-miR-124, PHMs/Cy5-miR-124, and Cy5-miR-124 was visualized by IVIS® spectrum *In Vivo* Imaging System (Caliper, MA, USA). Rats were anesthetized by administrating 1% (w/v) pentobarbital sodium solution to the abdomen and the optimized parameters (excitation, 640 nm; emission,700 nm) were set up for image acquisition at various time points. At 6 h after injection of the fluorescent complexes, internal organs (Heart, Liver, Spleen, Lung, and Kidney) and limbs were dissected and then visualized on the IVIS® spectrum system from Caliper Life Sciences (Hopkinton, MA, USA). The AIA rat treated with saline was set as a blank control. The normal rat treated with M-PHMs/Cy5-miR-124 was set as a negative control.

Assessment of Arthritis

Swelling of rat ankles was measured by a caliper and the severity of arthritis was graded via a macroscopic inspection and assessed by standard methods [8]. In short, the score for each ankle was defined as follows: 0=normal, 1=slight swelling and erythema, 2=mildest swelling and extended erythema, 3=moderate swelling and prolonged erythema, 4=severe swelling and extensive erythema. The highest score for arthritis was 16. Rats body weight was also measured as a means of a preliminary assessment of

systemic toxicity.

In vivo therapeutic efficacy

On the 9th day, rats were randomly divided into 6 groups of 6 animals each: Group I, untreated AIA model; Groups II-V, AIA rats were injected successively with M-PHMs/miR-124, M-PHMs/miR-124 NC, PHMs/miR-124, and MTX ; Group VI, normal control, healthy rat group without drug administration. The dose of MTX used was 38 μg/kg and the dose of miR-124 was 100 μg/kg. The formulations were given every other day via tail vein injection. The swelling of the ankles (right and left) was measured using a caliper every day. After the final treatment, radiological examination of the ankle on the hind paws was performed on a KODAK *in vivo* imaging system FX Pro from Carestream Health, Inc. (New Haven, CT, USA).

Biochemical Analysis

On day 18, blood samples were collected from the rat's orbit. Serum samples were then obtained by centrifuging blood at 3000 rpm for 10 min at 4℃. Concentrations of TNF-α, IL-6, IL-17, IL-18, IL-12, and IL-1β were determined by ELISA kits according to the manufacturer's instructions.

Histopathologic analysis

All rats were euthanized by 1% pentobarbital sodium solution on day 19 and ankle joints and livers were dissected in each group, fixed with 4% paraformaldehyde. Ankle joints were then decalcified in 20% (w/v) EDTA solution for 4 weeks for histopathologic analysis. The longitudinal sections were cut to 5 microns thick and stained with hematoxylin and eosin (H&E) and toluidine blue (TB). Synovial hyperplasia, cartilage destruction, and immune cell infiltration were observed under a microscope. To further evaluate the number of osteoclasts in the ankle, tartrate-resistant acid phosphatase (TRAP) staining was performed with a commercial kit and manufacturer's instructions were followed. The TRAP (+) cells number and TRAP (+) staining area were quantified by Image pro-plus 6.0.

Statistical analysis

Data were expressed as mean ± SEM and graphed by Origin 8.0. Statistical analysis of group differences and correlations were determined using the Student t-test. *$P < 0.05$ was considered statistically significant. **$P < 0.01$ and ***$P < 0.001$ were considered highly significant.

Reference

1. Xie J, Teng L, Yang Z, Zhou C, Liu Y, Yung BC, et al. A polyethylenimine-linoleic acid conjugate for antisense oligonucleotide delivery. Biomed Res Int. 2013; 2013: 710502.
2. Teng LS, Xie J, Teng LR, Lee RJ. Enhanced siRNA Delivery Using Oleic Acid Derivative of Polyethylenimine. Anticancer Res. 2012; 32: 1267-71.
3. Li H, Fu Y, Zhang T, Li Y, Hong X, Jiang J, et al. Rational Design of Polymeric Hybrid Micelles with Highly Tunable Properties to Co-Deliver MicroRNA-34a and Vismodegib for Melanoma Therapy. Adv Funct Mater. 2015; 25: 7457-69.

4. Li SD, Chen YC, Hackett MJ, Huang L. Tumor-targeted delivery of siRNA by self-assembled nanoparticles. Mol Ther. 2008; 16: 163-9.
5. Zhao J, Zhao M, Yu C, Zhang X, Liu J, Cheng X, et al. Multifunctional folate receptor-targeting and pH-responsive nanocarriers loaded with methotrexate for treatment of rheumatoid arthritis. Int J Nanomedicine. 2017; 12: 6735-46.
6. Bendele A. Animal models of rheumatoid arthritis. J Musculoskelet Neuronal Interact. 2001; 1: 377-85.
7. Carlson RP, Datko LJ, O'Neill-Davis L, Blazek EM, DeLustro F, Beideman R, et al. Comparison of inflammatory changes in established type II collagen- and adjuvant-induced arthritis using outbred Wistar rats. Int J Immunopharmacol. 1985; 7: 811-26.
8. Yang M, Ding J, Feng X, Chang F, Wang Y, Gao Z, et al. Scavenger Receptor-Mediated Targeted Treatment of Collagen-Induced Arthritis by Dextran Sulfate-Methotrexate Prodrug. Theranostics. 2017; 7: 97-105.

Supplementary data

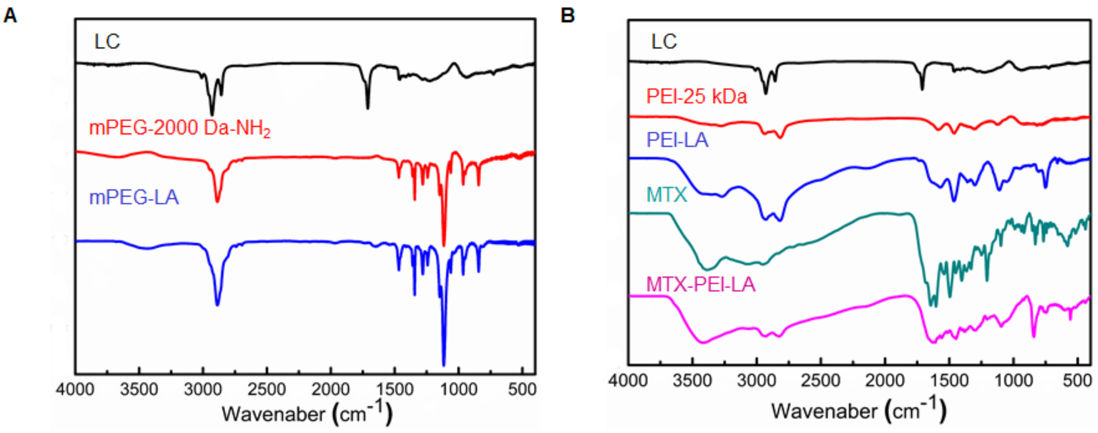

Figure S1. Fourier-transform infrared (FT-IR) spectra of LC, mPEG-2000 Da-NH_2, mPEG-LA, PEI-25 kDa, PEI-LA, MTX, and MTX-PEI-LA.

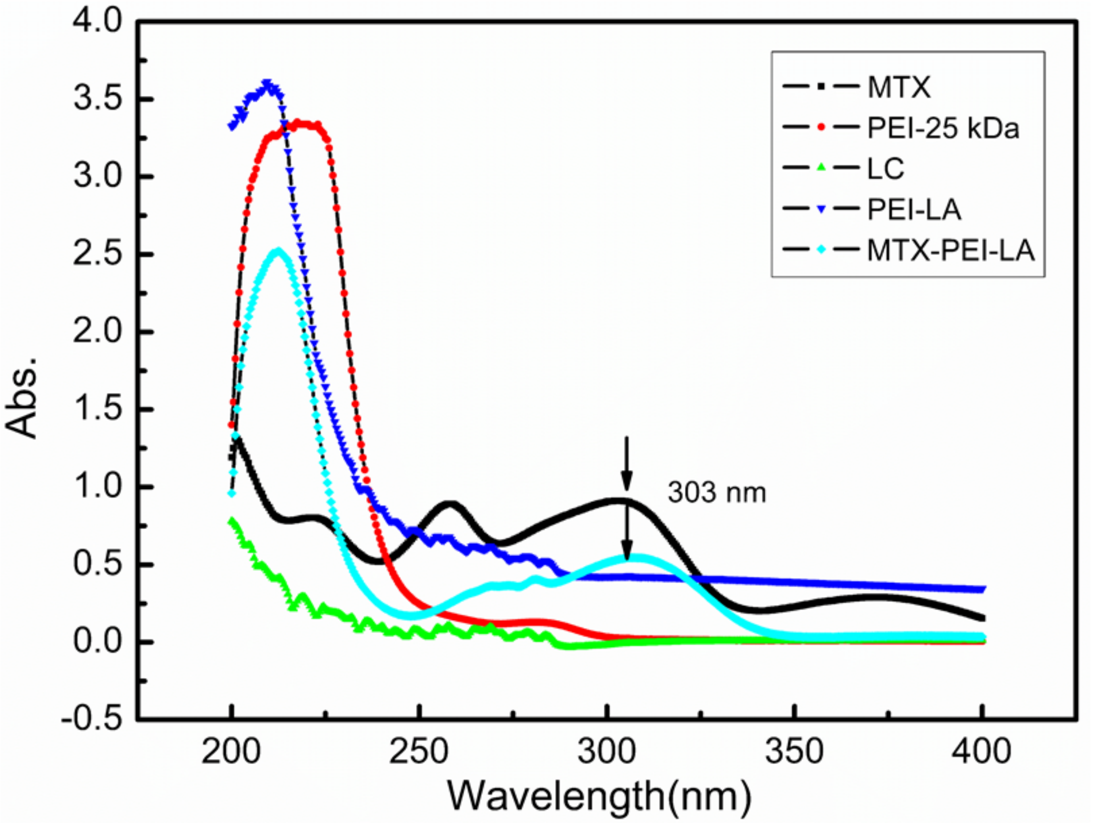

Figure S2. Absorption spectra of MTX, PEI, LC, PEI-LA, and MTX-PEI-LA.

Table S1. Size, polydispersity index (PDI) and zeta potential of M-PHMs with different weight percentage (%) of MTX-PEI-LA. Size, PDI, and zeta potential of various ratios of MTX-PEI-LA were measured by dynamic light scattering (DLS) on a Zeta Sizer Nano (n = 3, mean ± SEM).

Percentage (w/w) %	Diameter (nm)	PDI	Zeta potential (mv)
0	124.5±11.6	0.468±0.079	-7.31±0.81
2	136.5±2.0	0.256±0.011	-2.67±0.85
5	141.9±2.4	0.228±0.012	7.57±0.24
10	180.8±2.9	0.207±0.012	12.23±0.47
20	185.1±1.9	0.227±0.027	15.83±0.95
50	221.5±2.7	0.256±0.009	18.53±0.90
80	245.1±8.9	0.260±0.019	22.86±1.44
100	329.5±0.4	0.103±0.065	30.70±1.58

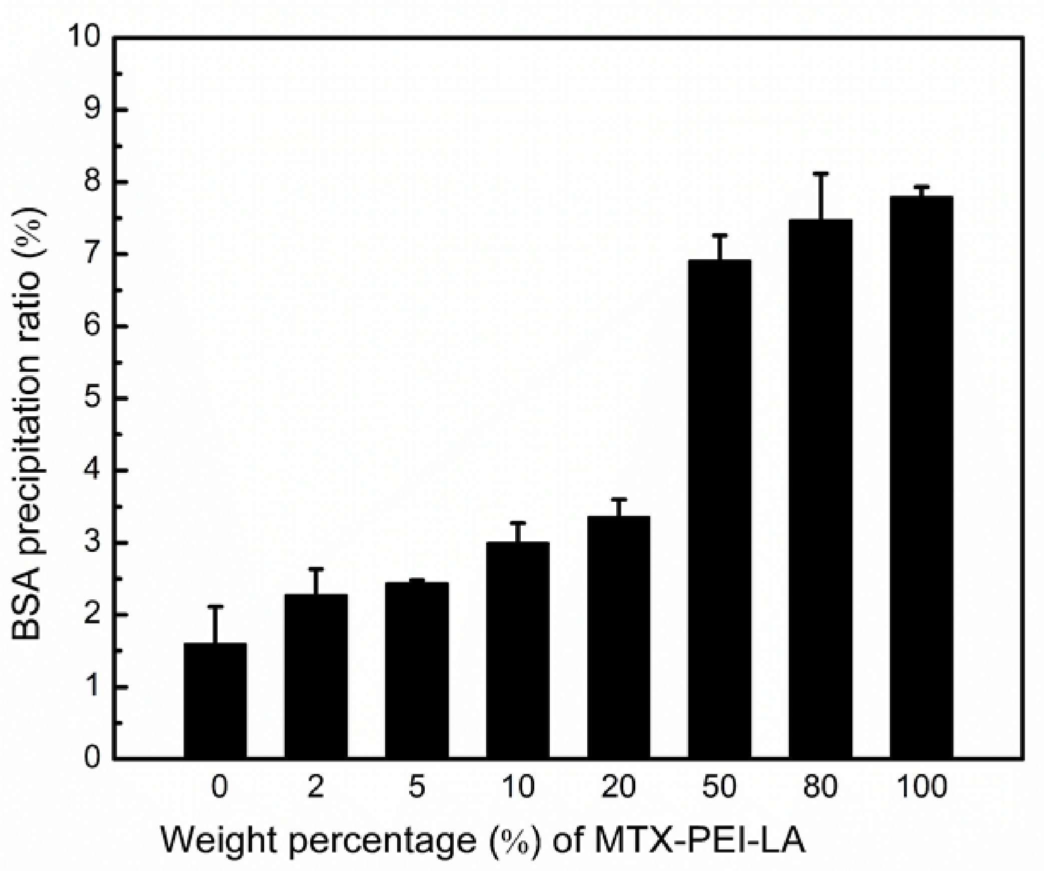

Figure S3. Colloidal stability analysis. BSA precipitation ratio (%) was applied to evaluate the colloidal stability of M-PHMs. BSA precipitation ratio (%) was obtained by incubating M-PHMs with different weight percentage (%) of MTX-PEI-LA with 10 mg/mL BSA solution (n=3, mean ± SEM).

Table S2. Hydrodynamic size, PDI and Zeta potential of M-PHMs/miR-124 and PHMs/miR-124. Size, PDI and Zeta potential of PHMs/miR-124 or M-PHMs/miR-124 (n=3, mean ± SEM) with N/P at 12/1 and 16/1. M-PHMs and PHMs consisted of the optimized percentage of the MTX-PEI-LA (5%, w/w).

Micelles	N/P	Size (nm)	Zeta potential (mV)	PDI
M-PHMs/miR-124	16/1	116.6±1.1	-4.66±0.20	0.217±0.008
PHMs/miR-124	12/1	112.7±3.9	4.77±1.85	0.202±0.001

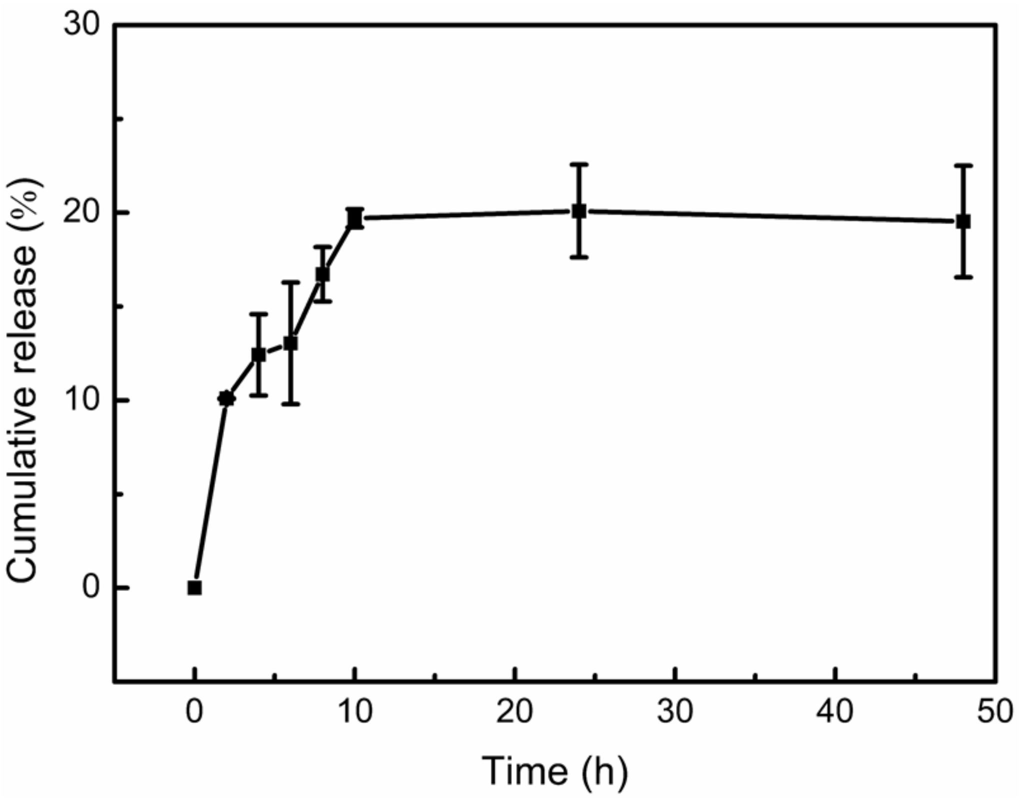

Figure S4. The release profile of miR-124. *In vitro* release profile of FAM-miR-124 in M-PHMs/FAM-miR-124 within 48 h (PBS, PH=7.4) (n=3, mean ± SEM).

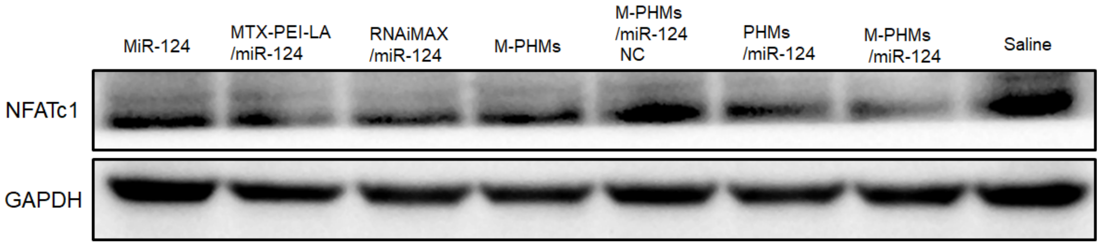

Figure S5. Inhibition of nuclear factor of activated T cells cytoplasmic 1 (NFATc1) protein expression by M-PHMs/miR-124. RAW 264.7 cells were separately treated with miR-124, MTX-PEI-LA/miR-124, RNAiMAX/miR-124, M-PHMs, M-PHMs/miR-124 NC, PHMs/miR-124, M-PHMs/miR-124, or saline. The cells were stimulated with 50 ng/mL receptor activator of the nuclear factor-κB (NF-κB) ligand (RANKL) for 48 h. NFATC1 expression levels were then measured by western blot assay.

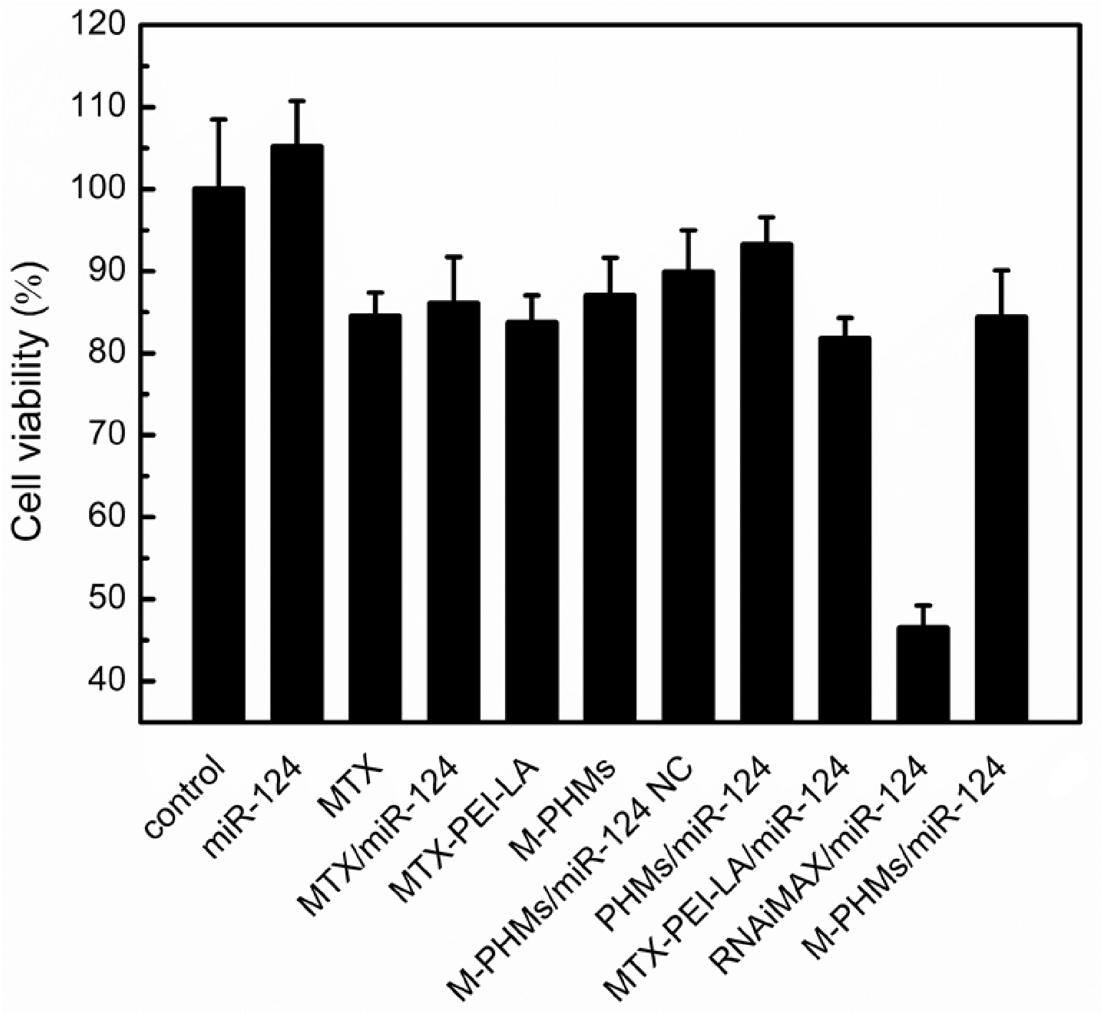

Figure S6. Cell viability in response to treatment with M-PHMs/Cy3-miR-124. RAW 264.7 cells were incubated in 96-well plate and activated by LPS for 48 h. The cell viability was obtained by MTT assay after incubation with miR-124, MTX, MTX and miR-124, MTX-PEI-LA, M-PHMs, M-PHMs/miR-124 NC, PHMs/miR-124, MTX-PEI-LA/miR-124, RNAiMAX/miR-124, or M-PHMs/miR-124 (MTX = 0.29 μg/mL, miR-124 = 50 nM) for 48 h. The untreated cells were set as the control. The values were mean ± SEM (n=6).

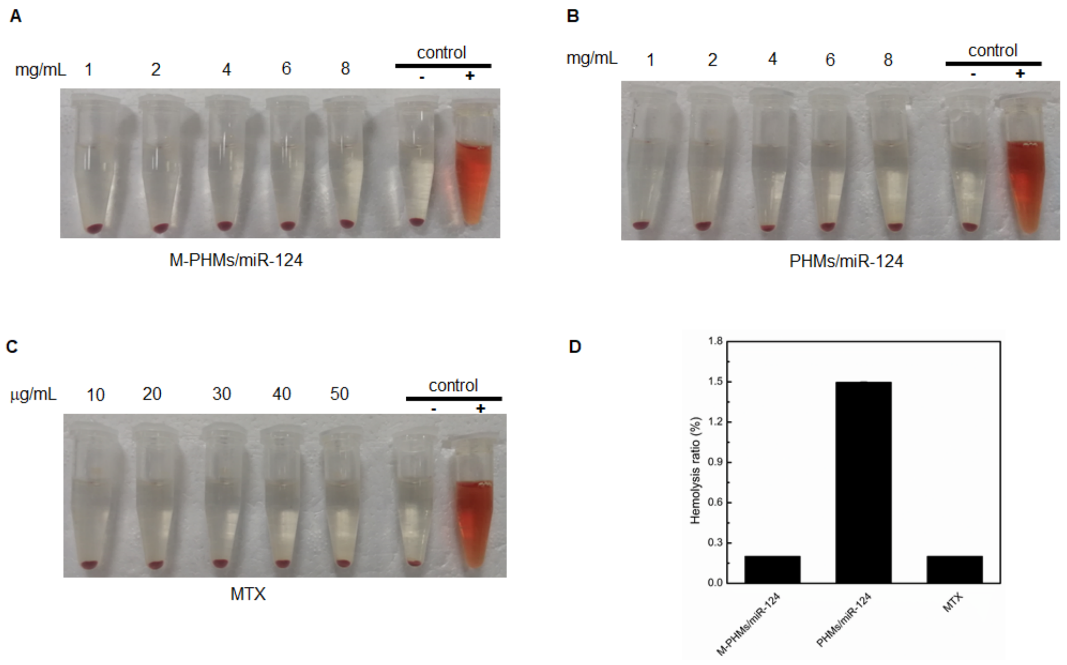

Figure S7. Hemolytic analysis. Supernatants of various concentrations of M-PHMs/miR-124 (A), PHMs/miR-124 (B), MTX (C) were visualized after incubated with 2% red blood cells standard dispersion (v/v) for 3 h. Saline and Triton X-100 were set up as the negative control (−) and positive control (+), respectively. (D) Hemolysis ratio was then evaluated and the values were mean ± SEM (n = 5).

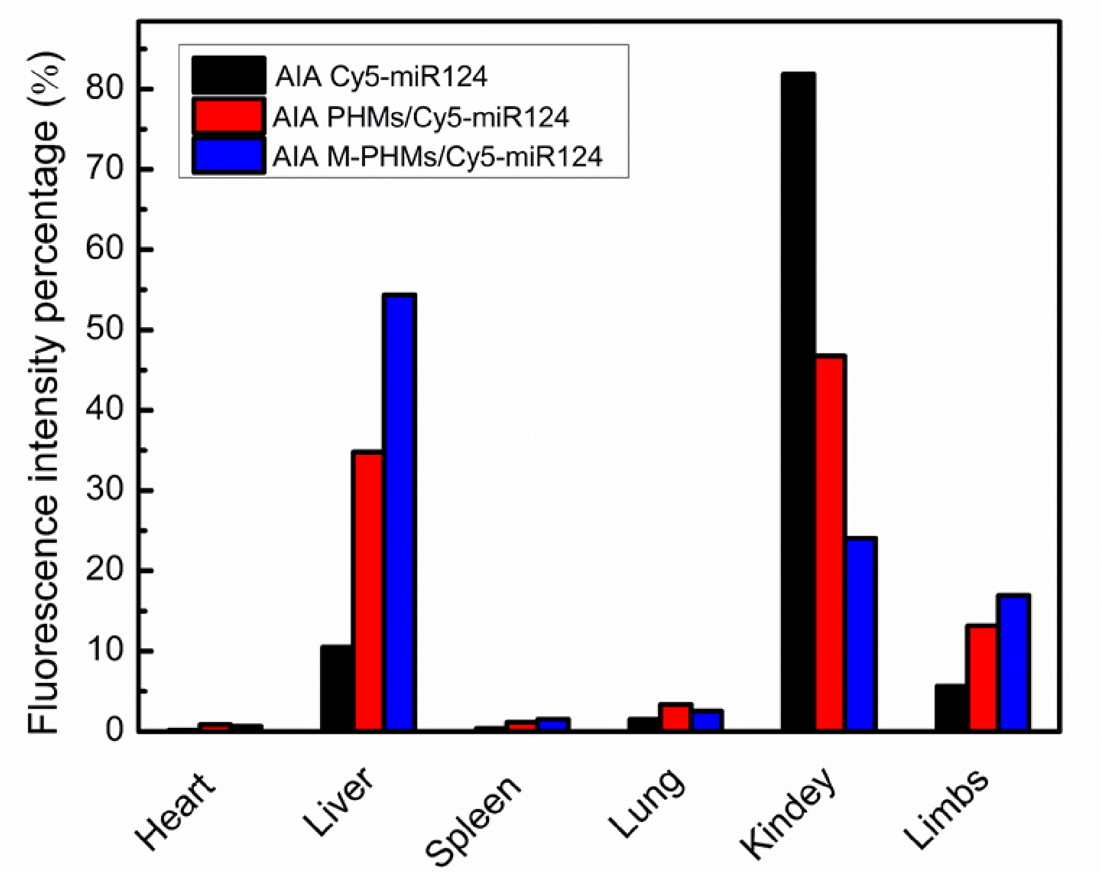

Figure S8. Fluorescence intensity percentage (%) in dissected organs and joints. Fluorescence intensity percentages (%) of organs and swollen joints treated with Cy5-miR-124, PHMs/Cy5-miR-124, and

M-PHMs/Cy5-miR-124 were quantified by In Vivo Imaging System at 6 h.

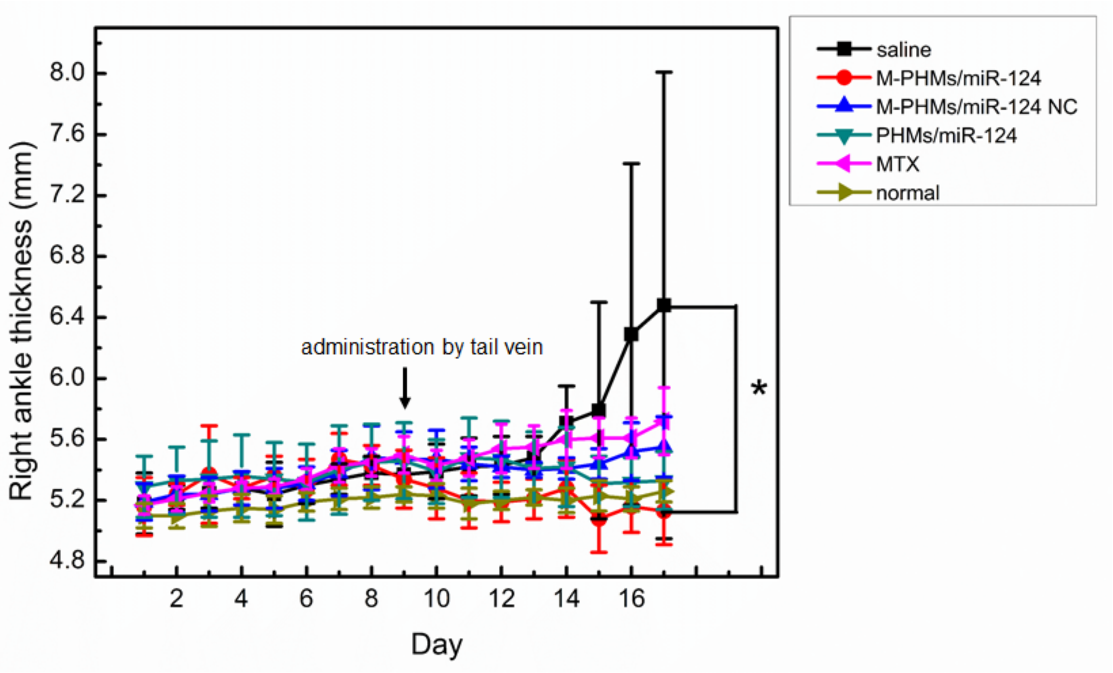

Figure S9. Analysis of right ankle swelling. Right ankle thickness of animals treated with various drug formulations was obtained (n=6, mean ± SEM, *P < 0.05, M-PHMs/miR-124group compared with AIA model group injected with saline).

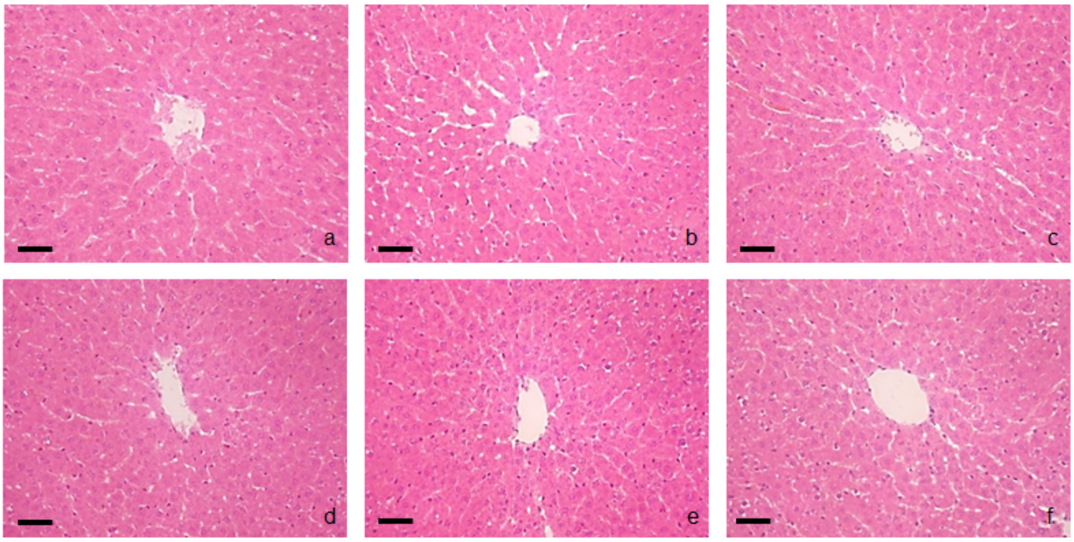

Figure S10. Histological analysis of the liver. Liver was excised from groups treated with saline as a negative control group (AIA, a), M-PHMs/miR-124 (b), M-PHMs/miR-124 negative control (c), PHMs/miR-124 (d), MTX (e), and normal rats as a blank control group (f) and was stained with HE. The scale bar in images was 100 μm.

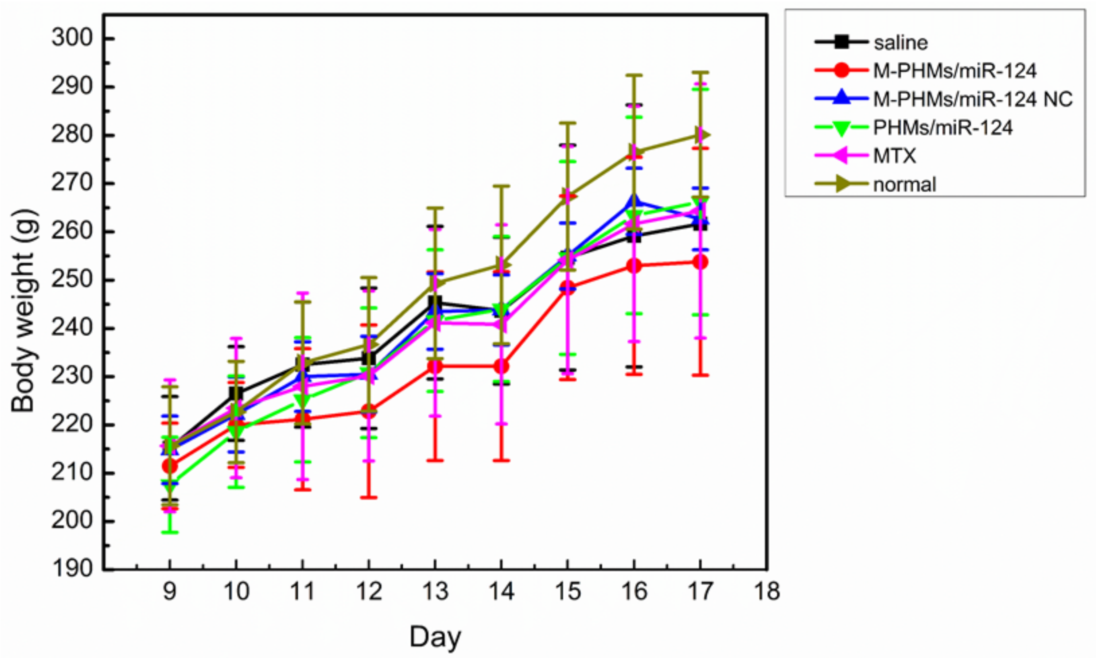

Figure S11. Evaluation of systemic toxicity. The body weight of the rats in different groups with varying formulations (saline, M-PHMs/miR-124, M-PHMs/miR-124 NC, PHMs/miR-124, and MTX). The values were mean ± SEM (n=6).

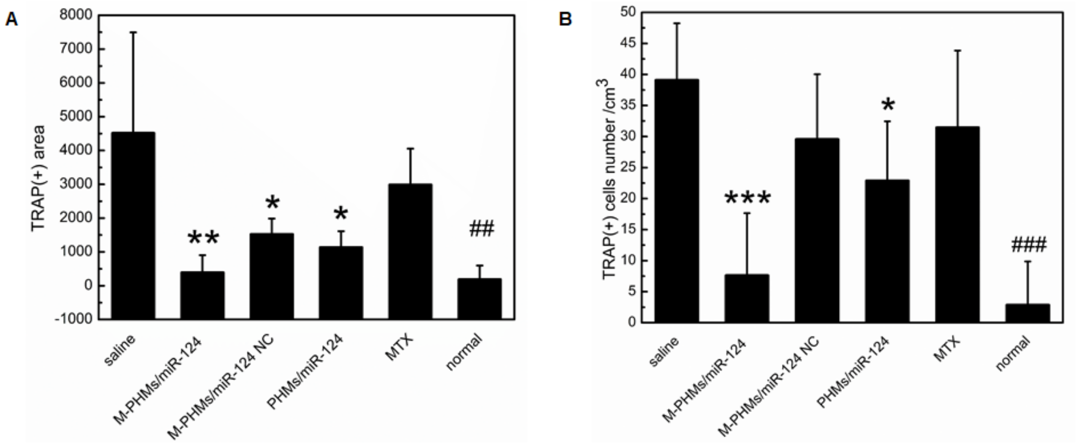

Figure S12. Quantitative analysis of TRAP (+) cells number and TRAP (+) area. TRAP (+) cells number (A) per cm^3 and TRAP (+) area (B) in images was analyzed using Image pro-plus 6.0 (n=6, mean ± SEM; *P < 0.05, **P < 0.01, ***P<0.001, M-PHMs/miR-124, M-PHMs/miR-124 NC, PHMs/miR-124, and MTX treated groups were separately compared with saline group; $^{\#}$P < 0.05, $^{\#\#}$P < 0.01, $^{\#\#\#}$P < 0.001, normal group compared with saline group).
